# Assessment of complementary health approaches use in pediatric oncology: Modification and preliminary validation of the "Which Health Approaches and Treatments Are You Using?" (WHAT) questionnaires

**DOI:** 10.1371/journal.pone.0294393

**Published:** 2024-03-06

**Authors:** Mohammad R. Alqudimat, Karine Toupin April, Lindsay Jibb, Charles Victor, Paul C. Nathan, Jennifer Stinson

**Affiliations:** 1 Lawrence S. Bloomberg Faculty of Nursing, University of Toronto, Toronto, Ontario, Canada; 2 Child Health Evaluation Sciences, Research Institute, The Hospital for Sick Children, Toronto, Ontario, Canada; 3 School of Rehabilitation Sciences and Department of Pediatrics, Children’s Hospital of Eastern Ontario Research Institute and Institute du Savoir Montfort, University of Ottawa, Ottawa, Ontario, Canada; 4 Institute of Health Policy, Management and Evaluation, University of Toronto, Toronto, Ontario, Canada; 5 Division of Haematology/Oncology, The Hospital for Sick Children, Toronto, Ontario, Canada; Public Library of Science, UNITED KINGDOM

## Abstract

**Objective:**

Complementary Health Approaches (CHA) are commonly used by children with cancer; however, a few health care providers (HCPs) inquire about the use of CHA. A standardized questionnaire could facilitate such clinical discussions. We aimed to adapt and determine the face and content validity of the “Which Health Approaches and Treatments are you using?" (WHAT) child and parent-report questionnaires in pediatric oncology.

**Methods:**

An electronic Delphi survey that included children with cancer (8–18 years), parents, and HCPs and CHA researchers was conducted to reach consensus on the content of the WHAT questionnaires in pediatric oncology. Children and parents from the Hospital for Sick Children (SickKids), and HCPs and researchers from the International Society of Pediatric Oncology and Pediatric Complementary and Alternative Medicine Research and Education Network completed the survey. To determine the face and content validity of the questionnaires, two iterative cycles of individual interviews were conducted with purposive samples of children (8–18 years), parents, and HCPs from SickKids.

**Results:**

Consensus was reached on all domains and items of the original WHAT questionnaires after one Delphi cycle (n = 61). For face and content validity testing, the first cycle of interviews (n = 19) revealed that the questionnaires were mostly comprehensive and relevant. However, the paper-based format of the original WHAT was not user-friendly, and generic items were vague and not aimed at facilitating clinical dialogues about CHA use. The WHAT questionnaires were then modified into electronic cancer-specific self- and proxy-report questionnaires including 13 and 15 items, respectively. The second cycle (n = 21) showed no need for further changes.

**Conclusions:**

The modified electronic cancer-specific WHAT questionnaires showed adequate face and content validity. The next step is to determine inter-rater reliability, construct validity, and feasibility of administration of the modified WHAT questionnaires in pediatric oncology.

## Introduction

Complementary health approaches (CHA) encompass health care products and practices that exist outside the mainstream healthcare system (i.e., biomedicine or conventional medicine), and include nutritional (e.g., dietary supplements and herbs), psychological (e.g., mindfulness and spiritual practices), and physical (e.g., massage and spinal manipulation) approaches, or combinations of these approaches (e.g., yoga and acupuncture) [1]. There is a lack of agreement in how to define and classify CHA [2–5]. One of the consequences of this variation in defining CHA is the inconsistency in how researchers measure and describe the patterns of CHA use [2, 3], which has contributed to the variation of the reported prevalence rates [3, 6–8]. The reported prevalence rate of CHA use in pediatric oncology ranges from 6% to 100% (median = 57.8%, n = 7,219 from 34 countries) [7].

The widespread use of CHA by children with cancer generates ethical, legal, and health-related concerns. Children with cancer usually use CHA in conjunction with conventional cancer treatments [6, 7, 9, 10]. This may be beneficial to alleviate cancer symptoms and side effects of cancer therapies; especially if the use is discussed with HCPs and supported by available evidence or clinical expertise. For instance, research has shown that acupuncture would be safe and effective for chemotherapy-induced nausea and vomiting [11–13]. Thus, HCPs should discuss these safe and effective CHA approaches with patients and families as they would discuss any other conventional treatments [14, 15]. Such discussions would help patients and families make informed (i.e., active or collaborative) decisions about using these CHA modalities [14, 16, 17]. However, assessment and discussion of CHA is not routine practice by pediatric oncology HCPs, which may be explained by their lack of knowledge about CHA [17–19], and their worries of potential conventional medicine abandonment as a result of CHA use for cancer care [20, 21]. Research shows that CHA use may delay a cancer diagnosis, and may lead to delayed receipt of or abandonment of conventional cancer treatments [10, 22–26], which may lead to significantly worse survival outcomes [20, 21]. Moreover, using some types of CHA (e.g., natural health products) may lead to a potential risk of interaction with conventional treatments leading to either greater toxicities or lower efficacy of cancer-targeted agents [27–30]. Thus, failure to disclose the use of such products can lead to preventable morbidity or even mortality.

The disclosure rates of CHA use by children with cancer are low in most countries ranging from 8% to 78% (median = 43%) based on data reported from 22 countries [7]. Children with cancer and their parents/caregivers may not voluntarily report information about CHA use unless HCPs explicitly ask [18, 31–36]. Moreover, children with cancer and their parents may feel hesitant to share this information unless HCPs show acceptance and openness to discuss the use of CHA [34–39]. Fear of doctor’s reaction to CHA use was reported in six studies as one of the reasons for not disclosing CHA use by children with cancer and their parents [10, 32, 40–43]. Therefore, it is crucial that HCPs be aware of CHA use, understand its use and its impact on the child’s health, and be prepared to initiate an open and non-judgmental discussions about CHA use. These discussions could ideally be facilitated by a questionnaire-based approach [44].

Our team published a recent systematic review of CHA questionnaires in pediatrics, and 35 questionnaires were identified [3]. None of these questionnaires were thoroughly validated, but the "Which Health Approaches and Treatments are you using?" (WHAT) questionnaires (versions for both children and parents) had evidence of initial validation for children with idiopathic arthritis (age 8–18 years) [44]. The WHAT were developed as paper-based short generic questionnaires. Stemming from this finding, we aimed to adapt and validate the WHAT questionnaires for use by oncology HCPs to facilitate open clinical discussions about CHA use with pediatric oncology patients, parents/caregivers, and their families; and assess CHA use by children with cancer 8–18 years of age (i.e., descriptive purpose). The final versions of the WHAT questionnaires must be relevant, comprehensive, and comprehensible but also easy to use by children with cancer (i.e., child self-report) and their parents (i.e., parent proxy-report) in clinical settings.

## Materials and methods

An adapted version of the Behavioural Model of Health Services Use [45] was used to conceptualize the underlying relationship between CHA use by children with cancer and the associated variables, according to the evidence (S1 Appendix). Both the child self- and parent proxy-report versions of the original WHAT questionnaires include four domains: child’s CHA use, variables associated with CHA use, perceived impact of CHA use, and communication about CHA within the family and with HCPs. The child self- and parent proxy-reports consist of 13 and 15 items, respectively [44].

### Design

In adapting the WHAT questionnaires, a Delphi survey was conducted (i.e., Phase 1). This survey was distributed electronically to children with cancer, parents, pediatric oncology HCPs, pediatric complementary HCPs, and CHA researchers. Phase 1 allowed consensus and adaption of the domains and items for use in pediatric oncology. Phase 2 explored the face and content validity of the questionnaires, assessing relevance, comprehensiveness, and comprehensibility in the context of pediatric oncology, according to the COnsensus-based Standards for the selection of health Measurement INstruments (COSMIN) guideline [46]. Iterative cycles of individual semi-structured interviews with children with cancer, their parents, and pediatric oncology HCPs were conducted using think-aloud and probing techniques. Interviews focused on identifying potential sources of response error by clarifying the item meaning (i.e., comprehensibility) and deciding on item relevance and questionnaires comprehensiveness [46–48]. We used Research Electronic Data Capture (REDCap) web-based application to collect data in both Phases [49]. The electronic data collection forms were pilot tested by two children with cancer, two parents, and two HCPs before starting on data collection. Ethical approval was obtained from the Research and Ethics Board at the Hospital for Sick Children (REB# 1000064764) and the Research and Ethics Boards at the University of Toronto (Protocol# 39414) before study commencement. Written informed consents/assents were obtained from patients and parents before participation in both Phases, and from HCPs before participation in Phase 2. Implied consents were received from HCPs and CHA researchers who participated in Phase 1.

#### Phase 1: Reaching consensus on the domains and items of the WHAT questionnaires

*Participants*. A convenience sample of children with cancer and one of their parents was invited to participate from the Hospital for Sick Children (SickKids). Patient eligibility included children diagnosed with cancer ≥ 3 months, English-speaking, 8–18 years of age, and having used at least one type of CHA since cancer diagnosis according to self- or proxy-report. English-speaking parents of such children were recruited. Exclusion criteria for child and parent participants were severe cognitive impairments or major comorbid illnesses that could preclude questionnaires completion as determined by their treating HCPs. Oncology HCPs were recruited through the International Society of Pediatric Oncology (SIOP). General pediatric and pediatric oncology HCPs, pediatric complementary HCPs and CHA researchers were recruited through the Pediatric Complementary and Alternative Medicine (CAM) Research and Education Network (PedCAM). Eligible HCPs and researchers had practiced in their field for at least one year. We targeted the same response rate achieved in the development study of the original WHAT questionnaires (i.e., 17%) [44]; and aiming for approximately equal numbers of participants from each sample group. Delphi cycles were ceased when consensus was reached on the domains and items of the WHAT questionnaires.

*Consensus rating scale*. Participants rated the importance of each domain and item of the questionnaires using a 1 to 9 scale according to the RAND/UCLA appropriateness method (RAM) [50]. Per the RAM, a rating of 1–3 was considered "inappropriate", 4–6 considered "uncertain", and 7–9 considered "appropriate". Consensus was achieved if the median rating for a domain or an item was "appropriate" (i.e., the domain or the item had to be retained) or "inappropriate" (i.e., to be removed). Consensus was not reached if the median rating was “uncertain” or there was disagreement. Disagreement among participants occurred when 30% or more rated the appropriateness of a domain or an item from 1–3 and 30% or more rated the same domain or item from 7–9 (i.e., polarized responses) [50]. If no consensus was reached, domains and items were moved to the subsequent cycle for re-rating.

*Procedure*. REDCap electronic surveys were sent via email to children and parents. For HCPs and CHA researchers, administrative approval from SIOP and PedCAM groups was received to invite their members. Leaders of these groups sent email invitations to group members, which described the study purpose and procedure, and directed participants to the REDCap survey. Children were asked to rate the importance of each domain and item of the self-report version, and parents were asked to rate the proxy-report. HCPs and researchers were asked to rate both versions of the WHAT questionnaires. We also asked all participants about the appropriate terminology that children and their parents would understand without ambiguity to refer to non-prescription health products and practices. Participants were asked to choose one phrase from a list of frequently used phrases in previous research, namely: Complementary and Alternative Medicine (CAM), CHA, Traditional and Complementary Medicine (TCM), or other. Finally, participants were asked to suggest modifications in the domains and items, as well as recommendations for domains and items that might be added and were then asked to complete a demographic survey.

*Data analysis*. Descriptive statistics were used to describe participant characteristics and their ratings of domains and items. The research team reviewed narrative responses to identify any suggestions to modify or add new domains or items.

#### Phase 2: Testing the face and content validity and modifying the WHAT questionnaires

*Participants selection*. A purposive sample of children with cancer, parents, and oncology HCPs were recruited from SickKids. We also recruited parents and children online through the Ontario Parents Advocating for Children with Cancer (OPACC) group via an email introduction by OPACC administrator. OPACC is a registered charity that is comprised of parents, guardians, survivors, and close relatives of children with cancer aiming to support children with cancer and their families across the province of Ontario, Canada. The same eligibility criteria were applied as in Phase 1. In Phase 2, we aimed to recruit for sample of children who were heterogeneous in terms of age (8–12 and 13–18 years old) and cancer diagnosis. The sample size was based on previous face and content validity testing research showing that two to three iterative cycles of cognitive interviews with a sample size of five to seven participants per cycle is sufficient [51–54].

*Procedure*. In-person or virtual individual interviews were conducted. Before the interview began, participants completed demographic and content validity ratio (CVR) forms using REDCap. The CVR form included questions that asked the participants to rate the importance of each item (i.e., relevance) using a 3-point scale (i.e., "essential", "useful but not essential", and "not necessary") [55]. During the interviews, children were asked to read over the child self-report, parents read the parent proxy-report. Children and parents were then asked to describe what they understood about the meaning of the item and whether it was easy to understand (comprehensibility), any essential items were missing (comprehensiveness), and all items and response options were relevant to the questionnaires’ purpose. HCPs were asked to review both versions of the WHAT questionnaires and to answer open-ended questions about item relevance and comprehensiveness of the questionnaires. There were no questions about comprehensibility since it could only be determined by questionnaire respondents (i.e., children and parents) [46]. All individual interviews were audio-recorded and transcribed verbatim for data analysis.

*Data analysis*. At the end of each cycle, qualitative and quantitative data were triangulated to determine evidence of face and content validity and to ensure the completeness of data by combining the results from different sources [56–58]. Triangulation was conducted by analyzing and combining qualitative (interview transcripts and field notes) and quantitative (CVR and percentage agreement) data. The audio-recorded transcripts and field notes were analyzed using simple descriptive content analysis method [59]. MA & BW conducted the data analysis by reviewing each transcript and its related field notes independently and identifying the frequency of participants’ concerns and perspectives about the content of the WHAT questionnaires. Disagreements were resolved by discussion. Descriptive statistics were used to describe participant characteristics. CVR was computed for each item using the following formula:

$$CVR=\frac{N_{e}-\;\frac{N}{2}}{\frac{N}{2}}$$

where, N_e_ is the number of participants indicating "essential" and N is the total number of participants [55]. If more than half of the participants rated an item as essential (i.e., positive CVR), the item was considered relevant [55]. Percent agreement was calculated to determine rating agreements between two groups: children and HCPs for the child version, and parents and HCP for the parent version. Good agreement was defined as at least 75% of participants rating an item as essential or useful [44]. Final decisions on modifying, removing, or retaining items in the modified WHAT were made by considering all findings from different data sources [44, 46].

## Results

### Phase 1: Reaching consensus on the domains and items of the WHAT questionnaires

#### Participants

The Delphi survey was completed by 61 participants with response rate per group as 68% (15/22) of children, 59% (13/22) of parents, 24% (10/41) of PedCAM pediatric HCPs and complementary HCPs and researchers, and 30% (23/77) of SIOP oncology HCPs. Table 1 shows the characteristics of the Delphi study participants.

**Table 1:** Characteristics of participants who participated in the Delphi survey.

	**Children with Cancer (n = 15)**	**Parents (n = 13)**
Age, mean (SD)	14.04 (3.1)	44.7 (5.2)
Sex, female n (%)	6 (40%)	12 (92%)
Educational Background, n (%)		
Grade 3–7 (8–12 years)	5 (33.3%)	—
Grade 8–12 (13–18 years)	10 (66.7%)	—
Graduated Secondary School	—	1 (7.7%)
Some College/Technical School	—	1 (7.7%)
Graduated College/Technical School	—	4 (30.7%)
Some Graduate School	—	1 (7.7%)
Graduate Degree	—	6 (46.2%)
Child’s Diagnosis, n (%)		
Acute Lymphoblastic Leukemia	4 (27%)	4 (30.7%)
Acute Myeloid Leukemia	2 (13%)	0
Brain Tumor	3 (20%)	3 (23.1%)
Neuroblastoma	1 (7%)	2 (15.4%)
Osteosarcoma	1 (7%)	0
Ewing’s Sarcoma	2 (13%)	2 (15.4%)
Other	2 (13%)	2 (15.4%)
Child’s disease duration in years, median (range)	1.25 (0.33–11.42)	1.33 (0.25–11.33)
Ethnicity, n (%)		
South Asian	1 (6.7%)	2 (15%)
South East Asian	1 (6.7%)	1 (7.7%)
White (Caucasian)	7 (46.6%)	7 (53.8%)
Other	3 (20%)	2 (15.4%)
Prefer not to answer	1 (6.7%)	1 (7.7%)
Missing data	2 (13.3%)	0
Country, n (%)		
Canada	15 (100%)	13 (100%)
	**Oncology HCPs (n = 21)**	**Complementary HCPs and researchers (n = 12)**
Age, mean (SD)	44.3 (12.3)	47.2 (12.1)
Sex, female n (%)	14 (67%)	5 (34%)
Profession, n (%)		
Pediatric Oncologist	8 (38.1%)	—
Pediatric Oncology Nurse	7 (33.3%)	—
Oncology Nurse Practitioner	3 (14.3%)	—
Other HCPs	3 (14.3%)	—
Naturopathic Practitioner	—	5 (41.6%)
Chiropractor	—	2 (16.7%)
Massage Therapist	—	2 (16.7%)
Other CHA HCPs and Researcher	—	3 (25%)
Years of experience, mean (SD)	19.67 (12.17)	19.08 (11.94)
Country, n (%)		
United States	7 (33%)	0
Canada	4 (19%)	10 (83%)
Kenya	3 (14%)	0
Turkey	2 (10%)	0
Cameroon	1 (5%)	0
Chile	1 (5%)	0
India	1 (5%)	0
Netherlands	1 (5%)	0
Uruguay	1 (5%)	0
Jordan	0	1 (8%)
China	0	1 (8%)

#### Delphi survey results

A total of 80.3% (49/61) of participants thought that the appropriate terms to refer to health products and practices that exists outside mainstream healthcare were CAM (28/49, 57%) or CHA (21/49, 43%). Only 16.4% (10/61) chose the term TCM, and 3.3% (2/61) suggested other terms (i.e., Holistic health modalities, over the counter, allied health partners). All domains and items were agreed upon by all participants and consensus was reached after one cycle of Delphi Survey (see Table 2).

**Table 2:** Median rating of each domain and items of the two versions of the WHAT questionnaires after one cycle of the Delphi survey.

**The Youth Version of the WHAT Questionnaire**	**Children with Cancer (n = 15)**			**Complementary HCPs and CHA Researchers (n = 12)**			**HCPs (n = 21)**			Percentage Agreement^c^	**Final Decision**
	Median	% ≥ 7^a^	% ≤ 3^b^	Median	% ≥ 7^a^	% ≤ 3^b^	Median	% ≥7^a^	% ≤ 3^b^		
**Domains**											
Child’s use of CHA (items 2, 3, 7, 7a, 8)	8	93%	0%	8	83%	8%	9	100%	0%	94%	Include domain
Factors associated with CHA use (items 5, 6, 7b)	8	80%	0%	8	92%	0%	9	90%	0%	88%	Include domain
Perceived impact of CHA use (items 7c, 7d, 7e)	8	93%	0%	8	83%	8%	9	100%	0%	94%	Include domain
Communication about CHA (items 1, 4)	8	87%	0%	8	52%	0%	9	95%	0%	92%	Include domain
**Sections and Items**											
Section 1: Conventional treatments	8	87%	7%	8.5	75%	8%	8	86%	0%	83%	Include section
Section 2: CHA definition and examples	8	93%	0%	7.5	75%	8%	9	95%	0%	90%	Include section
Item 1: Discussing CHA with health care team	8	100%	0%	8.5	48%	8%	9	95%	0%	94%	Include item
Item 2: Past use of CHA	8	93%	0%	8	67%	25%	8	90%	0%	85%	Include item
Item 3: Types of CHA used in the past	8	87%	0%	7.5	58%	17%	9	95%	0%	83%	Include item
Item 4: Discussing CHA with family	8	93%	0%	7.5	38%	8%	8	76%	0%	79%	Include item
Item 5: Person who decided to use CHA	8	93%	0%	7.5	58%	17%	8	81%	0%	79%	Include item
Item 6: Change on medical treatments because of CHA	8	80%	0%	8.5	92%	8%	9	90%	0%	88%	Include item
Item 7: Recent use of CHA (past two weeks)	8	87%	0%	8	75%	17%	9	86%	5%	83%	Include item
Item 7a: Types of CHA used recently	8	93%	0%	7.5	58%	17%	9	86%	5%	81%	Include item
Item 7b: Reasons of recent use of CHA	8	100%	0%	8	75%	0%	9	86%	5%	88%	Include item
Item 7c: How helpful was the recent use	8	100%	0%	8	83%	8%	8	81%	0%	88%	Include item
Item 7d: Benefits of recent use of CHA	8	93%	0%	8	75%	0%	8	76%	0%	81%	Include item
Item 7e: Risks/cons of recent use of CHA	8	87%	0%	8	67%	0%	8	81%	0%	79%	Include item
Item 8: Future use of CHA	8	80%	0%	8	83%	8%	8	81%	0%	81%	Include item
**The Parent Version of the WHAT Questionnaire**	**Parents (n = 13)**			**Complementary HCPs and CHA Researchers (n = 12)**			**HCPs (n = 21)**			**Percentage Agreement** ^c^	**Final Decision**
	Median	% ≥ 7^a^	% ≤ 3^b^	Median	% ≥ 7^a^	% ≤ 3^b^	Median	% ≥7^a^	% ≤ 3^b^		
**Domains**											
Child’s use of CHA (items 3, 4, 8, 8a, 9)	8	92%	0%	8	83%	8%	9	100%	0%	93%	Include domain
Factors associated with use of CHA (items 2, 6, 7, 8c)	8	92%	0%	8	92%	0%	9	90%	0%	91%	Include domain
Perceived impact of CHA use (items 8d, 8e, 8f)	8	85%	0%	8	83%	8%	9	100%	0%	91%	Include domain
Communication about CHA (items 1, 5, 8b)	8	92%	8%	8	52%	0%	9	95%	0%	93%	Include domain
**Sections and items**											
Section 1: Conventional treatments	9	85%	0%	9	83%	8%	8	90%	0%	87%	Include section
Section 2: CHA definition and examples	8	92%	0%	8	83%	8%	9	100%	0%	93%	Include section
Item 1: Discussing CHA with child’s HCP	9	92%	0%	8.5	48%	8%	9	95%	0%	91%	Include item
Item 2: Troubles getting CHA (i.e., access)	8	85%	8%	7	58%	0%	9	76%	10%	76%	Include item
Item 3: Child’s past use of CHA	8	85%	0%	8	67%	25%	9	100%	0%	87%	Include item
Item 4: Types of CHA used in the past by the child	9	85%	0%	7	58%	17%	9	95%	0%	83%	Include item
Item 5: Discussing CHA with the child	8	77%	0%	7.5	38%	8%	8	81%	0%	76%	Include item
Item 6: Person who decided on child’s use of CHA	8	85%	0%	7.5	58%	17%	8	81%	0%	89%	Include item
Item 7: Change on medical treatments because of CHA	9	85%	0%	8.5	92%	8%	9	90%	0%	85%	Include item
Item 8: Child’s recent use of CHA (past 2 weeks)	8	92%	0%	8	75%	8%	9	90%	10%	87%	Include item
Item 8a: Types of CHA used recently by the child	8	92%	0%	7.5	67%	8%	9	90%	10%	85%	Include item
Item 8b: Consult someone for child’s use of CHA	8	77%	0%	7.5	48%	0%	9	90%	0%	85%	Include item
Item 8c: Reasons of recent use of CHA by child	8	100%	0%	8	75%	0%	9	90%	5%	78%	Include item
Item 8d: How helpful was the child’s recent use	8	92%	0%	8	92%	8%	9	95%	0%	93%	Include item
Item 8e: Benefits of recent use of CHA by child	8	92%	0%	8	100%	0%	9	95%	0%	96%	Include item
Item 8f: Risks/cons of the child’s recent use	8	100%	0%	8	92%	0%	9	95%	0%	96%	Include item
Item 9: Future use of CHA by the child	9	92%	0%	8	83%	8%	8	81%	0%	85%	Include item

^a^ Percentage of participants who rated the domain or the item as more than or equal 7 on the scale from 1 to 9 (RAM method)

^b^ Percentage of participants who rated the domain or the item as less than or equal 3 on the scale from 1 to 9 (RAM method)

^c^ Percentage of participants from the three groups who rated the domain or the item as more than or equal to 7

### Phase 2: Testing face and content validity and modifying the WHAT questionnaires

#### Participants

Forty participants (12 children, 14 parents, 14 HCPs) completed two iterative cycles of individual interviews. The sample of children was heterogeneous in terms of age groups and cancer diagnosis. HCPs were experienced in the field with various professional backgrounds. Table 3 shows the characteristics of participants.

**Table 3:** Characteristics of participants in the face and content validity study.

**Children with Cancer**	**Cycle 1** (n = 5)	**Cycle 2** (n = 7)
Age, mean (SD)	13.45 (2.96)	12.56 (2.65)
Sex, female, n (%)	3 (60%)	4 (57%)
Educational Background, n (%)		
Grade 3–7	2 (40%)	3 (43%)
Grade 8–12	3 (60%)	4 (57%)
Child’s Diagnosis, n (%)		
Acute Lymphoblastic Leukemia	1 (20%)	2 (29%)
Acute Myeloid Leukemia	1 (20%)	0
Lymphoma	1 (20%)	1 (14%)
Neuroblastoma	0	1 (14%)
Osteosarcoma	0	2 (29%)
Ewing’s Sarcoma	2 (40%)	0
Other	0	1 (14%)
Mean disease duration in years, median (range)	4 (0.42–6.50)	0.75 (0.25–4.75)
Ethnicity, n (%)		
Aboriginal (Inuit, Metis, North American Indian)	1 (20%)	0
Arab/West Asian	2 (40%)	1 (14%)
South Asian	1 (20%)	1 (14%)
White (Caucasian)	2 (40%)	3 (43%)
Other	0	2 (29%)
Prefer not to answer	1 (20%)	0
**Parents of Children with Cancer**	**Cycle 1** (n = 7)	**Cycle 2** (n = 7)
Age, mean (SD)	43.14 (6.66)	43.98 (5.28)
Sex, female n (%)	6 (86%)	6 (86%)
Educational Background, n (%)		
Graduated Secondary School	1 (14.3%)	0
Some College/Technical School	0	1 (14.3%)
Graduated College/Technical School	1 (14.3%)	2 (28.6%)
Graduate Degree	5 (71.4%)	4 (57.1%)
Income, n (%)		
Less than $24,999	1 (14.3%)	1 (14.3%)
$25,000 to $49,999	2 (28.6%)	0
$75,000 to $99,999	1 (14.3%)	1 (14.3%)
$100,000 to $149,999	0	1 (14.3%)
$200,000 or more	0	1 (14.3%)
Prefer not to answer	3 (42.8%)	3 (42.8%)
Parents’ expectations of using CHA for their children, n (%)		
Using CHA would make my child feel better	3 (43%)	6 (86%)
Using CHA would prevent symptom of my child	2 (29%)	6 (86%)
Using CHA would cure my child’s illness	2 (29%)	2 (29%)
Using CHA would treat my child’s symptoms	2 (29%)	5 (71%)
Using CHA is safe	2 (29%)	7 (100%)
Other	1 (14%)	0
Parent’s use of CHA, Yes, n (%)	5 (71%)	4 (57%)
Child’s Diagnosis, n (%)		
Acute Lymphoblastic Leukemia	3 (42.8%)	1 (14.3%)
Acute Myeloid Leukemia	1 (14.3%)	0
Lymphoma	1 (14.3%)	1 (14.3%)
Brain Tumor	0	1 (14.3%)
Neuroblastoma	0	1 (14.3%)
Osteosarcoma	0	2 (28.5%)
Ewing’s Sarcoma	2 (28.6%)	0
Other	0	1 (14.3%)
Child’s disease duration in years, median (range)	4 (042–11.42)	0.58 (0.25–4.75)
Ethnicity, n (%)		
Aboriginal (Inuit, Metis, North American Indian)	1 (14.3%)	0
Arab/West Asian	2 (28.5%)	1 (14.3%)
South Asian	1 (14.3%)	1 (14.3%)
White (Caucasian)	1 (14.3%)	4 (57.1%)
Other	1 (14.3%)	1 (14.3%)
Prefer not to answer	1 (14.3%)	0
**Oncology HCPs**	**Cycle 1** (n = 7)	**Cycle 2** (n = 7)
Age, mean (SD)	52.29 (13.34)	40.14 (10.33)
Sex, Female n (%)	5 (71%)	7 (100%)
Profession, n (%)		
Pediatric oncologist	3 (42.8%)	1 (14.3%)
Pediatric oncology nurse practitioner	2 (28.6%)	1 (14.3%)
Master-prepared pediatric oncology nurse	0	3 (42.8%)
Pediatric oncology pharmacist	0	1 (14.3%)
Pediatric oncology fellow	2 (28.6%)	1 (14.3%)
Years of experience, mean (SD)	25.29 (12.51)	13.64 (9.39)
Discuss CHA with patients/parents, n (%)		
Never	0	0
Rarely	2 (28.6%)	4 (57.1%)
Sometimes	1 (14.3%)	1 (14.3%)
Most of the time	4 (57.1%)	0
Always	0	2 (28.6%)
Provide advice to patients/parents about CHA, n (%)		
Never	0	0
Rarely	2 (28.6%)	5 (71.4%)
Sometimes	3 (42.8%)	1 (14.3%)
Most of the time	2 (28.6%)	0
Always	0	1 (14.3%)

#### First cycle of the face and content validity testing

Five children, seven parents, and seven HCPs, who did not participate in Phase 1, completed the individual interviews. The interview duration was 30.9 minutes on average (SD = 7.5). Table 4 shows the findings of the first cycle of individual interviews. The participants confirmed that the WHAT questionnaires included comprehensive lists of questions. However, they did not find the paper format and the use of tables in the WHAT as user-friendly—limited space and a busy layout. A discussion within the research team concluded to redesign the WHAT as electronic versions, and to remove all tables and replace them with multiple choice and open-ended questions. Moreover, all items of the original WHAT questionnaires were meant to be generic. Many participants felt that the generic language of the questionnaires was vague and without time bound for many items. To minimize the ambiguity, participants suggested rephrasing the questions to be cancer-relevant and linking the timeframe to cancer events, like cancer diagnosis. Based on this, the research team decided to edit all items to be cancer specific. For item relevance, participants felt that the two versions of the WHAT included relevant questions about CHA use. However, a few items were not felt to be essential by some participants according to CVR scores (see Table 5). During the interviews, some HCPs stated that item five asked about information that could be “good to know” but not clinically essential. No other reasons for negative CVR were captured during the interviews from children and parents. The research team reviewed transcripts and CVR, and concluded that the generic nature of the questions might be the reason for the negative CVR scores. Thus, the research team decided to keep all items of both WHAT versions, with revision to be cancer-specific according to the comprehensibility testing results, and then verify their relevance with a new group of children, parents, and HCPs in the second cycle.

**Table 4:** Findings of the first cycle of the individual interviews regarding the comprehensiveness, comprehensibility, and relevance.

	Children n = 5	HCPs n = 7	Parents n = 7	Final Decision by the Research Team
Comprehensiveness	The child version was thought to be comprehensive. No extra items were suggested.	Both versions were thought to be comprehensive. Extra items were suggested by HCPs (each one came from a different HCP) to ask about: cannabis use, the difference between complementary and alternative approaches, parents’ use of CHA, and whether HCPs discuss CHA with children and parents.	The parent version was thought to be comprehensive. One parent suggested adding one item to ask about the cultural background.	Adding cannabis as an example of CHA to both versions of the WHAT is consistent with the purpose of the questionnaires because there is a trend of using cannabis by children with cancer [60], with limited information about its safety and efficacy. Other suggested items are irrelevant to the purpose of the WHAT as short clinical questionnaires, and they can be added to the WHAT instruction manual (i.e., instructions on how to use the WHAT questionnaires in clinical practice) as potential questions that HCPs would ask during discussions about CHA with children and parents.
Comprehensibility	The paper format and the use of tables in the WHAT were not user-friendly; for example, most children found section 1 was vague in terms of the timeline (past or current) and type of treatments (cancer-directed or other), and challenging to answer, given the large number of conventional therapies that had to be written in a small space in the table.	Most HCPs supported the idea that tables in both versions would be burdensome, and respondents might likely skip them.	The paper format and the use of tables in the WHAT were not user-friendly; for example, most parents found section 1 confusing about whether to include conventional therapies that children were on at home or receiving in the hospital and mentioned the concern about how difficult to answer this section was, given the large number of conventional therapies that had to be written in a small space in the table.	Redesign both versions of the WHAT as electronic versions. Remove all tables from both WHAT versions and replace them with multiple-choice and open-ended questions. Skip logic branching had also been applied, i.e., showing or hiding the follow-up questions based on answers to an earlier question, which would help minimize the respondents’ cognitive burden.
	The generic language of the questionnaires was vague and without time bound for many items.	Asking cancer-specific questions would help HCPs understand the complexities of CHA use in cancer.	The generic language of the questionnaires was vague and without time bound for many items.	Edit all items of both WHAT versions to be cancer specific.
	The purpose of the child version questionnaire was clear, except for two younger children, both 10 years of age. Most children were unfamiliar with the word “conventional,” and the two younger children could not understand most questions without help from their parents or researchers.	The purpose of both versions of the questionnaires was clear.	The purpose of the parent version questionnaire was clear. Most parents recommended modifying the word “conventional” with a word frequently used in clinical settings, like the primary health care team for cancer care.	Replace the vague words according to the participants’ suggestions. Test the comprehensibility of the child version in the next cycle with a sample of 2/7 (28.6%) young children (i.e., 8–11 years) to confirm the age group of the modified WHAT questionnaires.
Relevance	All items of the self-report were relevant. No explanation for the negative CVR (see Table 5 for CVR ratings).	All items were relevant (both versions). Item five asked about information that could be “good to know,” (see also Table 5 for CVR).	All items of the proxy-report were relevant. No explanation for the negative CVR (see Table 5 for CVR ratings).	Keep all items of both WHAT versions—according to data triangulation—with revision to be cancer specific and verify their relevance with a new group of children, parents, and HCPs in the second cycle.

**Table 5:** Agreement by participants regarding the relevance of each item of the WHAT questionnaires.

**The Youth Version of the Modified WHAT Questionnaire**	**Cycle 1**				**Cycle 2**				Final Decision
	Children n = 5	HCPs n = 7	Total n = 12	Percentage Agreement	Children n = 7	HCPs n = 7	Total n = 14	Percentage Agreement	
	CVR^a^	CVR^a^	n^b^	(%)^b^	CVR^a^	CVR^a^	n^b^	(%)^b^	
Section 1: Conventional treatments	0.6	0.71	11	92%	0.71	1.00	14	100%	Include section
Section 2: CHA definition and examples	0.6	1.00	12	100%	-0.14	1.00	14	100%	Include section
Item 1: CHA use since cancer diagnosis	0.2	0.71	12	100%	0.14	1.00	14	100%	Include item
Item 2: Used types of CHA by the child since cancer diagnosis	-0.6	1.00	12	100%	0.71	1.00	14	100%	Include item
Item 3: Discussion between Patients and HCP about CHA use	-0.2	0.43	12	100%	0.14	0.71	14	100%	Include item
Item 4: Who decided that the child should use CHA	-0.2	0.71	12	100%	0.43	0.71	14	100%	Include item
Item 5: Discussion between patients and parents about CHA use	-1	-0.43	11	92%	-0.14	0.43	13	93%	Include item
Item 6: Have the child changed cancer treatment for CHA use	0.2	0.71	12	100%	0.14	0.71	13	93%	Include item
Item 7: CHA use during the past four weeks (recent use)	-1	0.43	12	100%	0.14	0.71	14	100%	Include item
Item 7a: Used types of CHA by the child in the past four weeks	0.2	0.71	12	100%	0.43	0.43	14	100%	Include item
Item 7b: Reasons of recent use of CHA by the child	0.2	-0.14	12	100%	0.43	0.71	14	100%	Include item
Item 7c: How helpful was the recent use of CHA	-0.2	1.00	12	100%	0.43	0.71	14	100%	Include item
Item 7d: What were the benefit of recent use of CHA	0.6	1.00	12	100%	0.43	0.71	13	93%	Include item
Item 7e: What were the risks of the recent use of CHA	0.6	1.00	12	100%	0.71	1.00	13	93%	Include item
Item 8: Plans for future use of CHA by the child	-0.2	0.43	11	92%	0.43	0.71	14	100%	Include item
**The Parent Version of the Modified WHAT Questionnaire**	**Cycle 1**				**Cycle 2**				Final Decision
	Parents n = 7	HCPs n = 7	Total n = 14	Percentage Agreement	Parents n = 7	HCPs n = 7	Total n = 14	Percentage Agreement	
	CVR^a^	CVR^a^	n^b^	(%)^b^	CVR^a^	CVR^a^	n^b^	(%)^b^	
Section 1: Conventional Cancer treatments	0.71	0.71	13	93%	1.00	0.71	14	100%	Include section
Section 2: CHA definition and examples of common CHA used in Pediatric Oncology	0.43	1.00	14	100%	0.43	0.71	14	100%	Include section
Item 1: Child’s CHA use since cancer diagnosis	0.71	1.00	14	100%	0.14	0.71	14	100%	Include item
Item 2: Used types of CHA by the child since cancer diagnosis	0.71	1.00	14	100%	0.71	0.71	14	100%	Include item
Item 3: Discussion between Parents and HCP about CHA use	0.43	0.71	14	100%	0.43	0.71	14	100%	Include item
Item 4: Who decided that the child should use CHA	0.43	0.71	14	100%	0.14	0.71	14	100%	Include item
Item 5: Discussion between patients and parents about CHA use	0.14	-0.71	13	93%	0.43	0.14	14	100%	Include item
Item 6: Have Parents changed cancer treatment for CHA use	0.71	0.71	14	100%	0.43	0.43	14	100%	Include item
Item 7: Difficulty accessing CHA	0.43	0.14	14	100%	0.14	0.14	13	93%	Include item
Item 8: Child’s CHA use during the past four weeks (recent use)	0.71	0.71	14	100%	0.43	0.43	14	100%	Include item
Item 8a: Used types of CHA by the child in the past four weeks	0.71	0.71	13	93%	0.71	0.43	14	100%	Include item
Item 8b: Did Parents consult someone to use CHA for the child	0.43	0.71	14	100%	-0.14	0.43	13	93%	Include item
Item 8c: Reasons of recent use of CHA by the child	0.43	0.43	14	100%	0.14	0.43	13	93%	Include item
Item 8d: How helpful was the recent use of CHA	1.00	0.71	14	100%	0.14	0.71	14	100%	Include item
Item 8e: What were the benefit of recent use of CHA	1.00	0.71	14	100%	0.71	0.71	13	93%	Include item
Item 8f: What were the risks of the recent use of CHA	1.00	0.71	14	100%	0.43	1.00	13	93%	Include item
Item 9: Plans for future use of CHA by the child	0.71	0.43	14	100%	0.43	0.71	14	100%	Include item

CVR ratings of generic items and modified cancer-specific items of the questionnaires are presented under cycle 1 and cycle 2 columns, respectively.

^a^CVR > 0 is considered positive CVR which means more than half of the sample rated the item as “essential”

^b^Number and percentage of participants who found the item to be essential or useful. Good percentage agreement being defined as at least 75% rated an item as “essential” or “useful”

#### Resulting modified WHAT questionnaires

Based on the results of the first cycle of the face and content validity testing, the research team modified the items of the original WHAT questionnaires and designed electronic versions using REDCap, which would ideally be integrated into the electronic health record application for use clinically. The modified self- (13 items, S2 Appendix) and proxy-report (15 items, S3 Appendix) versions of the WHAT were cancer-specific and asked about the use of CHA based on three timeframes: since cancer diagnosis, in the past four weeks, and future use [49]. The research team also added examples to make the questions easier to complete. CHA terminology, definition, and classification from the US National Center for Complementary and Integrative Health (NCCIH) were adapted, which were the most cited in the past research [1, 3]; and using the term “CHA” was consistent with our Delphi findings.

#### Second cycle of the face and content validity testing

A new sample of seven children, seven parents, and seven HCPs completed the individual interviews. The same procedure as the first cycle was followed. The interview duration was 25.4 minutes on average (SD = 7.7). Table 6 shows the findings of the second cycle of individual interviews. All participants confirmed that the questionnaires included comprehensive short lists of questions for clinical use. All children and parents understood all items of the modified WHAT questionnaires except two children (8 and 9 years old) who could not independently read, understand, or answer all questions without help from parents or the researcher. So, the research team changed the targeted age group for the self-report version and let parents help young children (i.e., 8 to 11 years) complete the child version. The two versions of the modified WHAT questionnaires were determined by all participants to represent relevant questions on CHA use in pediatric oncology (see Table 5 for CVR).

**Table 6:** Findings of the second cycle of the individual interviews regarding the comprehensiveness, comprehensibility, and relevance.

	Children n = 7	HCPs n = 7	Parents n = 7	Final Decision by the Research Team
Comprehensiveness	The child version was thought to be comprehensive and included short lists of questions for clinical use and would be sufficient to describe the use of CHA by children with cancer. No extra items were suggested.	Both versions were thought to be comprehensive and included short lists of questions for clinical use and would be sufficient to describe the use of CHA by children with cancer. No extra items were suggested	The parent version was thought to be comprehensive and included short lists of questions for clinical use and would be sufficient to describe the use of CHA by children with cancer. One parent suggested adding one item to ask about CHA frequency, but the same parent mentioned that it would add an extra burden on the respondents.	The research team discussed the suggestion by the parent and decided that this item would be difficult to measure (i.e., too precise to be measured in a brief clinical questionnaire) and it could be added to the instruction manual as a potential point of discussion by HCPs with children and their parents during clinic visits.
Comprehensibility	The purpose of the child version questionnaire was clear, except for two younger children, 8 and 9 years old. All children understood all items of the child version of the modified WHAT questionnaires except the two younger children. No suggestions were received to revise any item.	The purpose of both versions of the questionnaires was clear. No suggestions were received to revise any item.	The purpose of the parent version questionnaire was clear. All parents understood all items of the parent version of the modified WHAT questionnaires. No suggestions were received to revise any item.	For the child self-report version, parents or caregivers can help young children (8 to 11 years) complete the questionnaire. Older children (12 to 18 years) would complete the self-report independently. No need for further changes.
Relevance	All items were relevant. No explanation for the negative CVRs (see Table 5 for CVR ratings).	All items in both versions were relevant (see Table 5 for CVR ratings).	All items were relevant. For item 8b (negative CVR ratings, see Table 5), during the interviews, most parents felt that this item would be important since this would reflect whether the recent use of CHA was based on recommendations from a reliable source.	Keep all modified items of both versions of the WHAT questionnaires according to data triangulation. No need for further changes.

## Discussion

This article presents the results of adapting and testing the face and content validity of the WHAT questionnaires for multidimensional clinical assessment of CHA use in pediatric oncology. We introduce the first electronic disease-specific CHA questionnaires (i.e., the modified WHAT) that have evidence of face and content validity and are designed to assess CHA use and initiate clinical discussions about CHA in pediatric oncology. The lack of such CHA questionnaires was considered a gap as reported in previous systematic reviews [3, 6, 7]. Also, previous research has supported the need for open dialogue in pediatric oncology settings about CHA and the resultant need to assess the use of CHA using a questionnaire [17–19, 33, 44]. Open and non-judgemental discussion about CHA use in clinical settings would be a key step to enhance disclosure rates, minimize the potential risk of interactions with conventional treatments, and enhance HCPs understanding of CHA use and its impact on the child’s health. Ultimately, adapted and tailored assessment questionnaires will enrich HCPs knowledge about CHA, identify safe and effective CHA products and practices, and may pave the way toward integrative pediatric oncology care.

Results from the Delphi survey showed that most participants (80%) were familiar with the terms CAM and CHA. Accordingly, we used the term CHA in the modified WHAT questionnaires to refer to the products and practices that children with cancer may use on their own or be recommended by their parents or caregivers without medical prescription. We also included a section to define CHA with examples of the frequently used CHA products and practices by children with cancer. Including friendly terminology and a clear definition of CHA should help respondents understand the subsequent questions about CHA, and ultimately minimize cognitive burden associated with questionnaire completion and response errors. Also, we retained the section about conventional treatments, simplified it, and modified its wording to ask about the prescribed therapies by primary health care team for cancer care (i.e., oppose to the generic format), so respondents would actively distinguish between CHA and conventional cancer treatments.

We engaged children, parents, and HCPs in refining and testing the face and content validity of the modified WHAT questionnaires. This has not been the case for all existing pediatric oncology CHA questionnaires [3, 8]. Engaging children and parents in designing patient-reported outcome questionnaires is essential to capture their perspectives on whether the included items are relevant to the construct of interest and in the context of use. Also, the child’s and the parent’s perspectives are vital to determine whether the questionnaire is comprehensive and easily understood by respondents [46, 61, 62]. Moreover, using a sequential phased approach ensured iterative adjustments of the WHAT questionnaires and testing the face and content validity by children, parents, and HCPs ensured the inclusion of essential domains and items that are consistent with the measurement purpose of the modified WHAT questionnaires.

In this research project, we adapted pediatric generic short CHA questionnaires and used their contents as the core sets of domains and items in the Delphi survey. We also modified them into electronic disease-specific questionnaires in the face and content validity study. This is consistent with a previously recommended method of developing and validating an adult oncology CHA questionnaire [63]. Researchers suggested designing a standardized CHA questionnaire to assess CHA use across adult populations, which would help researchers generate a meaningful comparison among different populations by using a core set of questions (i.e., generic) that could be adapted and refined later as disease-specific questionnaires, like for cancer and arthritis [63]. The results of our first cycle of the face and content validity testing revealed that the generic items of the questionnaires were vague and not meaningful for children with cancer and their parents. HCPs highlighted that asking cancer-specific questions would help HCPs understand the complexities of CHA use in cancer, as opposed to the general use of CHA. Therefore, we refined all items to be cancer-specific to enhance the comprehensibility and offer questionnaires that would help HCPs describe CHA use in pediatric oncology. Also, electronic questionnaires versions were found to be more user-friendly and flexible, especially for some complex questions. Although the main purpose of the modified WHAT questionnaires is to facilitate HCPs discussion about CHA with children and parents, the collected data could also be used for research purposes and enable standardized comparison between pediatric oncology findings and other studies in pediatric conditions using the original generic WHAT versions.

Most of the existing pediatric oncology CHA questionnaires were designed to be used as parent-proxy report [3, 8]. We have now generated face and content validity tested self- and proxy-report CHA questionnaires for use in clinical pediatric oncology settings. A self-report questionnaire will support direct understanding of child’s CHA use from the child and can facilitate their active participation in discussions with HCPs. It can be argued that parent proxy-report questionnaires can be acceptable, especially as parents are routinely asked by HCPs to comment on their child’s health status. Agreement between the self- and proxy-report is not granted unless confirmed by inter-rater reliability testing; for example, previous research has shown that proxy-report results are not equivalent to the self-report in a study on perceptions of quality of life in children under chiropractic care [64]. Therefore, the two versions of the modified WHAT could be used to compare the responses on self- and proxy-reports about CHA use in pediatric oncology, which was not explored previously.

Comparable to the original development study of the WHAT questionnaires, we used the COSMIN checklist to guide refining and initially validating the two versions of the WHAT questionnaires [44, 46]. This was used to ensure a rigorous testing process, which was not the case with most CHA questionnaires across pediatric populations [3, 8]. Contrary to most existing CHA questionnaires, we also identified the measurement purpose of the modified WHAT questionnaires, identified the conceptual framework, and engaged a sample of the target population.

### Limitations

The CVR rating was not easy to understand by most children, especially the younger ones. Therefore, there were discrepancies between CVR ratings and what children mentioned during the interviews about how important each item was, which led to discrepancies with HCPs’ CVR ratings for a few items. We analyzed the findings using multiple data sources to develop a comprehensive understanding about the face and content validity concerns and decide upon the inclusion of items in the modified questionnaires. Also, we used the percentage of agreement between groups of participants, which has helped to quantify the agreement between participating groups to ensure a rigorous assessment of content validity.

In Phase 2, children, parents, and oncology HCPs were recruited to determine the face and content validity of the WHAT. This was because we aimed to test the face and content validity of the WHAT by a sample of the populations who will use the WHAT in clinical settings: the respondents (children and parents), and data users (oncology HCPs who will use the data to initiate clinical discussion). However, including complementary HCPs in future research would enrich our understanding about CHA use and enhance the content of the modified WHAT questionnaires. We were also not able to recruit participants from pediatric oncology newly arrived immigrant populations in Canada; thus, future research should be conducted to ask for their perspectives, and tailor the WHAT content to their CHA use experience. This is crucial as the WHAT could be used to enrich HCPs knowledge about the use of CHA by these importation populations. Finally, further research needs to be conducted in the context of pediatric oncology in low-middle-income countries before the WHAT can be utilized in these countries as clinical CHA questionnaires.

## Conclusions

We present the first steps of adapting and testing the face and content validity of two versions of clinical CHA questionnaires to be used in pediatric oncology. Children, parents, and HCPs agreed on the content of both versions of the WHAT questionnaires after one cycle of Delphi survey. Two iterative cycles of individual interviews and modifications made to the WHAT questionnaires according to suggestions from children, parents and HCPs have helped ensure adequate face and content validity for use in pediatric oncology. The next steps of the validation process will include determining the interrater reliability, construct validity, and the feasibility of administration of the modified versions of the WHAT questionnaires among children with cancer and their parents. Clinical utility testing of the modified WHAT is another planned step prior will be able to use it in clinical settings. The modified WHAT questionnaires will then help HCPs assess and document CHA use steadily and initiate communication and knowledge exchange about the benefits and risks of CHA between families and HCPs, which ultimately may enhance the disclosure rate, minimize the risk of potential CHA-conventional treatment interaction, and ensure informed decision making about using CHA. The routine use of the modified WHAT questionnaires may help HCPs and researchers to determine what approaches are being used and which may be helpful, thus leading to the development of a new CHA research agenda for pediatric oncology.

## Supporting information

S1 Appendix Conceptual model. (PDF)

S2 Appendix The child version of the modified WHAT questionnaire. (PDF)

S3 Appendix The parent version of the modified WHAT questionnaire. (PDF)

## References

[1] NCCIH. Complementary, Alternative, or Integrative Health_ What’s In a Name?: NCCIH; 2022 [July 17]. https://nccih.nih.gov/health/integrative-health.

[2] Fennell D, Liberato AS, Zsembik B. Definitions and patterns of CAM use by the lay public. Complement Ther Med. 2009;17(2):71–7. Epub 2009/02/03. doi: 10.1016/j.ctim.2008.09.002 . 19185264

[3] Alqudimat MR, Toupin April K, Hundert A, Jibb L, Victor C, Nathan PC, et al. Questionnaires assessing the use of complementary health approaches in pediatrics and their measurement properties: A systematic review. Complement Ther Med. 2020;53:102520. Epub 2020/10/18. doi: 10.1016/j.ctim.2020.102520 . 33066855

[4] Kristoffersen AE, Fonnebo V, Norheim AJ. Use of complementary and alternative medicine among patients: classification criteria determine level of use. J Altern Complement Med. 2008;14(8):911–9. Epub 2008/11/08. doi: 10.1089/acm.2008.0127 . 18990042

[5] Gaboury I, Toupin April K, Verhoef M. A qualitative study on the term CAM: is there a need to reinvent the wheel?. Complementary and Alternative Medicine. 2012;12:13. 22909051 10.1186/1472-6882-12-131 PMC3462712

[6] Bishop FL, Prescott P, Chan YK, Saville J, von Elm E, Lewith GT. Prevalence of complementary medicine use in pediatric cancer: a systematic review. Pediatrics. 2010;125(4):768–76. Epub 2010/03/24. doi: 10.1542/peds.2009-1775 . 20308209

[7] Diorio C, Lam CG, Ladas E, Njuguna F, Afungchwi GM, Taromina K, et al. Global Use of Traditional and Complementary Medicine in Childhood Cancer: A Systematic Review. Journal of Global Oncology. 2017;3(6):791–800. Epub 2017/12/16. doi: 10.1200/JGO.2016.005587 . 29244989 PMC5735959

[8] Toupin April K, Moher D, Stinson J, Byrne A, White M, Boon H, et al. Measurement properties of questionnaires assessing complementary and alternative medicine use in pediatrics: a systematic review. PLoS One. 2012;7(6):e39611. Epub 2012/07/07. doi: 10.1371/journal.pone.0039611 . 22768098 PMC3387262

[9] Luthi E, Diezi M, Danon N, Dubois J, Pasquier J, Burnand B, et al. Complementary and alternative medicine use by pediatric oncology patients before, during, and after treatment. BMC Complement Med Ther. 2021;21(1):96. Epub 2021/03/20. doi: 10.1186/s12906-021-03271-9 . 33736643 PMC7977159

[10] Al-Qudimat MR, Rozmus CL, Farhan N. Family strategies for managing childhood cancer: using complementary and alternative medicine in Jordan. J Adv Nurs. 2011;67(3):591–7. Epub 2010/12/02. doi: 10.1111/j.1365-2648.2010.05517.x . 21118291

[11] Gottschling S, Reindl Tk Fau—Meyer S, Meyer S Fau—Berrang J, Berrang J Fau—Henze G, Henze G Fau—Graeber S, Graeber S Fau—Ong MF, et al. Acupuncture to alleviate chemotherapy-induced nausea and vomiting in pediatric oncology—a randomized multicenter crossover pilot trial. Klin Padiatr. 2008;220(6):365–70. doi: 10.1055/s-0028-1086039 18949672

[12] Reindl TK, Geilen W, Hartmann R, Wiebelitz KR, Kan G, Wilhelm I, et al. Acupuncture against chemotherapy-induced nausea and vomiting in pediatric oncology. Interim results of a multicenter crossover study. Supportive care in cancer: official journal of the Multinational Association of Supportive Care in Cancer. 2006;14(2):172–6. Epub 2005/07/16. doi: 10.1007/s00520-005-0846-z . 16021478

[13] Chokshi SK, Ladas EJ, Taromina K, McDaniel D, Rooney D, Jin Z, et al. Predictors of acupuncture use among children and adolescents with cancer. Pediatr Blood Cancer. 2017;64(7). Epub 2017/02/09. doi: 10.1002/pbc.26424 . 28176457

[14] McClafferty H, Vohra S, Bailey M, Brown M, Esparham A, Gerstbacher D, et al. Pediatric Integrative Medicine. Pediatrics. 2017;140(3). Epub 2017/08/30. doi: 10.1542/peds.2017-1961 . 28847978

[15] Gilmour J, Harrison C Fau—Asadi L, Asadi L Fau—Cohen MH, Cohen Mh Fau—Vohra S, Vohra S. Informed consent: advising patients and parents about complementary and alternative medicine therapies. Pediatrics. 2011;128(4):1098–4275 (Electronic). doi: 10.1542/peds.2010-2720H 22045862

[16] Gagnon EM, Recklitis CJ. Parents’ decision-making preferences in pediatric oncology: the relationship to health care involvement and complementary therapy use. Psychooncology. 2003;12(5):442–52. Epub 2003/07/02. doi: 10.1002/pon.655 . 12833557

[17] Stub T, Quandt SA, Kristoffersen AE, Jong MC, Arcury TA. Communication and information needs about complementary and alternative medicine: a qualitative study of parents of children with cancer. BMC Complement Med Ther. 2021;21(1):85. Epub 2021/03/10. doi: 10.1186/s12906-021-03253-x . 33685422 PMC7938468

[18] Al-Omari A, Al-Qudimat M, Abu Hmaidan A, Zaru L. Perception and attitude of Jordanian physicians towards complementary and alternative medicine (CAM) use in oncology. Complement Ther Clin Pract. 2013;19(2):70–6. Epub 2013/04/09. doi: 10.1016/j.ctcp.2013.01.002 . 23561063

[19] Valji R, Adams D, Dagenais S, Clifford T, Baydala L, King WJ, et al. Complementary and alternative medicine: a survey of its use in pediatric oncology. Evid Based Complement Alternat Med. 2013;2013:527163. Epub 2013/12/07. doi: 10.1155/2013/527163 . 24307910 PMC3836302

[20] Hord JD, Rehman W, Hannon P, Anderson-Shaw L, Lou Schmidt M. Do Parents Have the Right to Refuse Standard Treatment for Their Child With Favorable-Prognosis Cancer? Ethical and Legal Concerns. Journal of Clinical Oncology 2006;24(34):5454–6. doi: 10.1200/JCO.2006.06.4709 17135648

[21] Cohen M. Legal and Ethical Issues Relating to Use of Complementary Therapies in Pediatric Hematology/Oncology. Complementary and Alternative Medicine. 2006;28(3):190–3. doi: 10.1097/01.mph.0000210401.28685.57 16679948

[22] Friedrich P, Lam CG, Kaur G, Itriago E, Ribeiro RC, Arora RS. Determinants of Treatment Abandonment in Childhood Cancer: Results from a Global Survey. PLoS One. 2016;11(10):e0163090. Epub 2016/10/14. doi: 10.1371/journal.pone.0163090 . 27736871 PMC5063311

[23] Borker AS, Chaudhary N. Pattern of refusal to treat and abandonment in a new pediatric oncology unit in Southern India. Pediatr Blood Cancer. 2012;59(6):1100. Epub 2012/09/19.

[24] Ariffin H, Abdullah WA, de Bruyne J, Lee CL, Peng LH. Belief in traditional healers amongst Malaysian parents of children with cancer. J Trop Pediatr. 1997;43:375–6. 10.1093/tropej/43.6.375 9476465

[25] Yeh C, Tsai J, Li W, Chen H, Lee S, Lin C, et al. Use of alternative therapy among pediatric oncology patients in Taiwan. Pediatr Hematol Oncol 2000;17:55–65. doi: 10.1080/088800100276668 10689715

[26] Martel D, Bussieres JF, Theoret Y, Lebel D, Kish S, Moghrabi A, et al. Use of alternative and complementary therapies in children with cancer. Pediatr Blood Cancer. 2005;44(7):660–8. Epub 2005/02/17. doi: 10.1002/pbc.20205 . 15714446

[27] Kelly KM. Bringing evidence to complementary and alternative medicine in children with cancer: Focus on nutrition-related therapies. Pediatr Blood Cancer. 2008;50(2 Suppl):490–3; discussion 8. Epub 2007/12/08. doi: 10.1002/pbc.21402 . 18064645

[28] Lanski SL, Greenwald M, Perkins A, Simon HK. Herbal Therapy Use in a Pediatric Emergency Department Population: Expect the Unexpected. Pediatrics. 2003;111(5):981–5. 12728075 10.1542/peds.111.5.981

[29] Jankovic M, Spinetta J, Martins A, Pessio A, Sullivan M, D’Angio G, et al. Non-Conventional Therapies in Childhood Cancer: Guidelines for Distinguishing Non-Harmful From Harmful Therapies: A Report of the SIOP Working Committee on Psychosocial Issues in Pediatric Oncology. Pediatr Blood Cancer. 2004;42:106–8. doi: 10.1002/pbc.10418 14752802

[30] Goldman RD, Rogovik AL, Lai D, Vohra S. Potential interactions of drugnatural health products and natural health products-natural health products among children. J Pediatr 2008;152(4):521–6, 6 e–4. doi: 10.1016/j.jpeds.2007.09.026 18346508

[31] Hsu J, Gordon C, Megason G. Complementary and alternative medicine use in pediatric hematology/oncology patients at the University of Mississippi. Pediatr Blood Cancer 2013:60:S84.

[32] Dhanoa A, Yong TL, Yeap SJ, Lee IS, Singh VA. Complementary and alternative medicine use amongst Malaysian orthopaedic oncology patients. BMC Complement Altern Med. 2014;14:404. Epub 2014/10/18. doi: 10.1186/1472-6882-14-404 . 25324121 PMC4209028

[33] Kemper KJ, Vohra S, Walls R. The use of complementary and alternative medicine in pediatrics. Pediatrics. 2008;122(6):1374–86. Epub 2008/12/03. doi: 10.1542/peds.2008-2173 . 19047261

[34] Jenkins BN, Vincent N, Fortier MA. Differences in referral and use of complementary and alternative medicine between pediatric providers and patients. Complement Ther Med. 2015;23(3):462–8. Epub 2015/06/09. doi: 10.1016/j.ctim.2015.02.004 . 26051582

[35] Chen C, Chong YJ, Hie SL, Sultana R, Lee SHD, Chan WSD, et al. Complementary and alternative medicines use among pediatric patients with epilepsy in a multiethnic community. Epilepsy Behav. 2016;60:68–74. Epub 2016/05/15. doi: 10.1016/j.yebeh.2016.04.008 . 27179195

[36] Beattie JF, Thompson MD, Parks PH, Jacobs RQ, Goyal M. Caregiver-reported religious beliefs and complementary and alternative medicine use among children admitted to an epilepsy monitoring unit. Epilepsy Behav. 2017;69:139–46. Epub 2017/03/13. doi: 10.1016/j.yebeh.2017.01.026 . 28285234

[37] Adams D, Dagenais S, Clifford T, Baydala L, King WJ, Hervas-Malo M, et al. Complementary and alternative medicine use by pediatric specialty outpatients. Pediatrics. 2013;131(2):225–32. Epub 2013/01/16. doi: 10.1542/peds.2012-1220 . 23319526

[38] Naja F, Alameddine M, Abboud M, Bustami D, Al Halaby R. Complementary and alternative medicine use among pediatric patients with leukemia: the case of Lebanon. Integr Cancer Ther. 2011;10(1):38–46. Epub 2010/11/10. doi: 10.1177/1534735410384591 . 21059622

[39] Magi T, Kuehni CE, Torchetti L, Wengenroth L, Luer S, Frei-Erb M. Use of Complementary and Alternative Medicine in Children with Cancer: A Study at a Swiss University Hospital. PLoS One. 2015;10(12):e0145787. Epub 2015/12/24. doi: 10.1371/journal.pone.0145787 . 26694320 PMC4687920

[40] Isaac-Otero G, Molina-Alonso D, Asencio-Lopez L, Leal-Leal C. The use of alternative or complementary treatment in pediatric oncologic patients: Survey of 100 cases in a level III attention institute. Gac Med Mex. 2016;152:5–9. 26927638

[41] Sandar K, Win Lai M, Han W. Use of complementary and alternative medicine (CAM) in children with cancer at Yangon Children’s Hospital. Myanmar Health Sciences Research Journal 2013;25:183–8.

[42] Suen YI, Gau BS, Chao SC. Survey of parents of children with cancer who look for alternative therapies [in Chinese]. Ju Li Zhi. 2005;52:29–38. 15986299

[43] Njuguna F, Mostert S, Seijffert A, Musimbi J, Langat S, van der Burgt RH, et al. Parental experiences of childhood cancer treatment in Kenya. Supportive care in cancer: official journal of the Multinational Association of Supportive Care in Cancer. 2015;23(5):1251–9. Epub 2014/10/17. doi: 10.1007/s00520-014-2475-x . 25318695

[44] Toupin April K, Stinson J, Boon H, Duffy CM, Huber AM, Gibbon M, et al. Development and Preliminary Face and Content Validation of the "Which Health Approaches and Treatments Are You Using?" (WHAT) Questionnaires Assessing Complementary and Alternative Medicine Use in Pediatric Rheumatology. PLoS One. 2016;11(3):e0149809. Epub 2016/03/11. doi: 10.1371/journal.pone.0149809 . 26964088 PMC4786318

[45] Andersen RM. Revisiting the behavioral model and access to medical care: does it matter? Journal of Health and Social Behavior 1995;36(1):1–10. 7738325

[46] Terwee CB, Prinsen CAC, Chiarotto A, Westerman MJ, Patrick DL, Alonso J, et al. COSMIN methodology for evaluating the content validity of patient-reported outcome measures: a Delphi study. Qual Life Res. 2018;27(5):1159–70. Epub 2018/03/20. doi: 10.1007/s11136-018-1829-0 . 29550964 PMC5891557

[47] Hofmeyer A, Sheingold BH, Taylor R. Do You Understand What I Mean? How Cognitive Interviewing Can Strengthen Valid, Reliable Study Instruments And Dissemination Products. Journal of International Education Research 2015;11(4):261–7.

[48] Boeije H, Willis G. The Cognitive Interviewing Reporting Framework (CIRF). Methodology. 2013;9(3):87–95. doi: 10.1027/1614-2241/a000075

[49] Harris PA, Taylor R, Thielke R, Payne J, Gonzalez N, Conde JG. Research electronic data capture (REDCap)—a metadata-driven methodology and workflow process for providing translational research informatics support. J Biomed Inform. 2009;42(2):377–81. Epub 2008/10/22. doi: 10.1016/j.jbi.2008.08.010 . 18929686 PMC2700030

[50] Fitch K, Bernstein SJ, Aguilar MD, Burnand B, LaCalle JR, Lazaro P, et al. The RAND/UCLA Appropriateness Method User’s Manual. Santa Monica, CA: RAND Corporation; 2001. https://www.rand.org/pubs/monograph_reports/MR1269.html. Also available in print form.

[51] Molich R. A critique of “how to specify the participant group size for usability studies: A practitioner’s guide by Macefield. Journal of Usability Studies, 2010. 5(3): p. 124–128.

[52] Macefield R. How to specify the participant group size for usability studies: A practitioner’s guide. Journal of Usability Studies. 2009;5(1):34–45.

[53] Stinson JN, Jibb LA, Nguyen C, Nathan PC, Maloney AM, Dupuis LL, et al. Development and testing of a multidimensional iPhone pain assessment application for adolescents with cancer. J Med Internet Res. 2013;15(3):e51. Epub 2013/03/12. doi: 10.2196/jmir.2350 . 23475457 PMC3636147

[54] Jibb LA, Cafazzo JA, Nathan PC, Seto E, Stevens BJ, Nguyen C, et al. Development of a mHealth Real-Time Pain Self-Management App for Adolescents With Cancer: An Iterative Usability Testing Study. J Pediatr Oncol Nurs. 2017;34(4):283–94. Epub 2017/04/06. doi: 10.1177/1043454217697022 . 28376666

[55] Gilbert GE, Prion S. Making Sense of Methods and Measurement: Lawshe’s Content Validity Index. Clinical Simulation in Nursing. 2016;12:530–1. doi: 10.1016/j.ecns.2016.08.002

[56] Casey D, Murphy K. Issues in using methodological triangulation in research. Nurse Res. 2009;16(4):40–55. Epub 2009/08/06. doi: 10.7748/nr2009.07.16.4.40.c7160 . 19653545

[57] Bekhet AK, Zauszniewski JA. Methodological Triangulation: An Approach to Understanding Data. Nurse Researcher. 2012;20(2):40–3. doi: 10.7748/nr2012.11.20.2.40.c9442 23316537

[58] Carter N, Bryant-Lukosius D, DiCenso A, Blythe J, Neville AJ. The use of triangulation in qualitative research. Oncol Nurs Forum. 2014;41(5):545–7. Epub 2014/08/28. doi: 10.1188/14.ONF.545-547 . 25158659

[59] Sandelowski M. Focus on Research Methods—Whatever Happened to Qualitative Description? Research in Nursing & Health. 2000;23:334–40. 10940958 10.1002/1098-240x(200008)23:4<334::aid-nur9>3.0.co;2-g

[60] Chapman S, Protudjer J, Bourne C, Kelly LE, Oberoi S, Vanan MI. Medical cannabis in pediatric oncology: a survey of patients and caregivers. Supportive care in cancer: official journal of the Multinational Association of Supportive Care in Cancer. 2021;29(11):6589–94. Epub 2021/05/02. doi: 10.1007/s00520-021-06202-z . 33932156

[61] Prinsen CAC, Mokkink LB, Bouter LM, Alonso J, Patrick DL, de Vet HCW, et al. COSMIN guideline for systematic reviews of patient-reported outcome measures. Qual Life Res. 2018;27(5):1147–57. Epub 2018/02/13. doi: 10.1007/s11136-018-1798-3 . 29435801 PMC5891568

[62] Mokkink LB, de Vet HCW, Prinsen CAC, Patrick DL, Alonso J, Bouter LM, et al. COSMIN Risk of Bias checklist for systematic reviews of Patient-Reported Outcome Measures. Qual Life Res. 2018;27(5):1171–9. Epub 2017/12/21. doi: 10.1007/s11136-017-1765-4 . 29260445 PMC5891552

[63] Quandt S, Verhoef MJ, Arcury T, Lewith GT, Steinsbekk A, Kristoffersen AE, et al. Development of an International Questionnaire to Measure Use of Complementary and Alternative Medicine (I-CAM-Q). The Journal of Alternative and Complementary Medicine. 2009;15(4):331–9. doi: 10.1089/acm.2008.0521 19388855 PMC3189003

[64] Alcantara J, Ohm J, Alcantara J. Comparison of pediatric self reports and parent proxy reports utilizingPROMIS: Results from a chiropractic practice-based research network. Complementary Therapies in Clinical Practice 2017;29:48–52. doi: 10.1016/j.ctcp.2017.08.003 29122268

