# Supplementary material for: Assessment of complementary health approaches use in pediatric oncology: Modification and preliminary validation of the "Which Health Approaches and Treatments Are You Using?" (WHAT) questionnaires
Source: PLoS One. 2024 Mar 6;19(3):e0294393. doi: 10.1371/journal.pone.0294393 (PMC10917275; doi:10.1371/journal.pone.0294393)
Supplement: S1 Appendix — (PDF) [file pone.0294393.s001.pdf]

Supporting information

S1 Appendix. Conceptual Model

An adapted version of the “Behavioural Model of Health Services Use” * was used to conceptualize the underlying relationship between CHA use by children with cancer and associated variables, according to the evidence

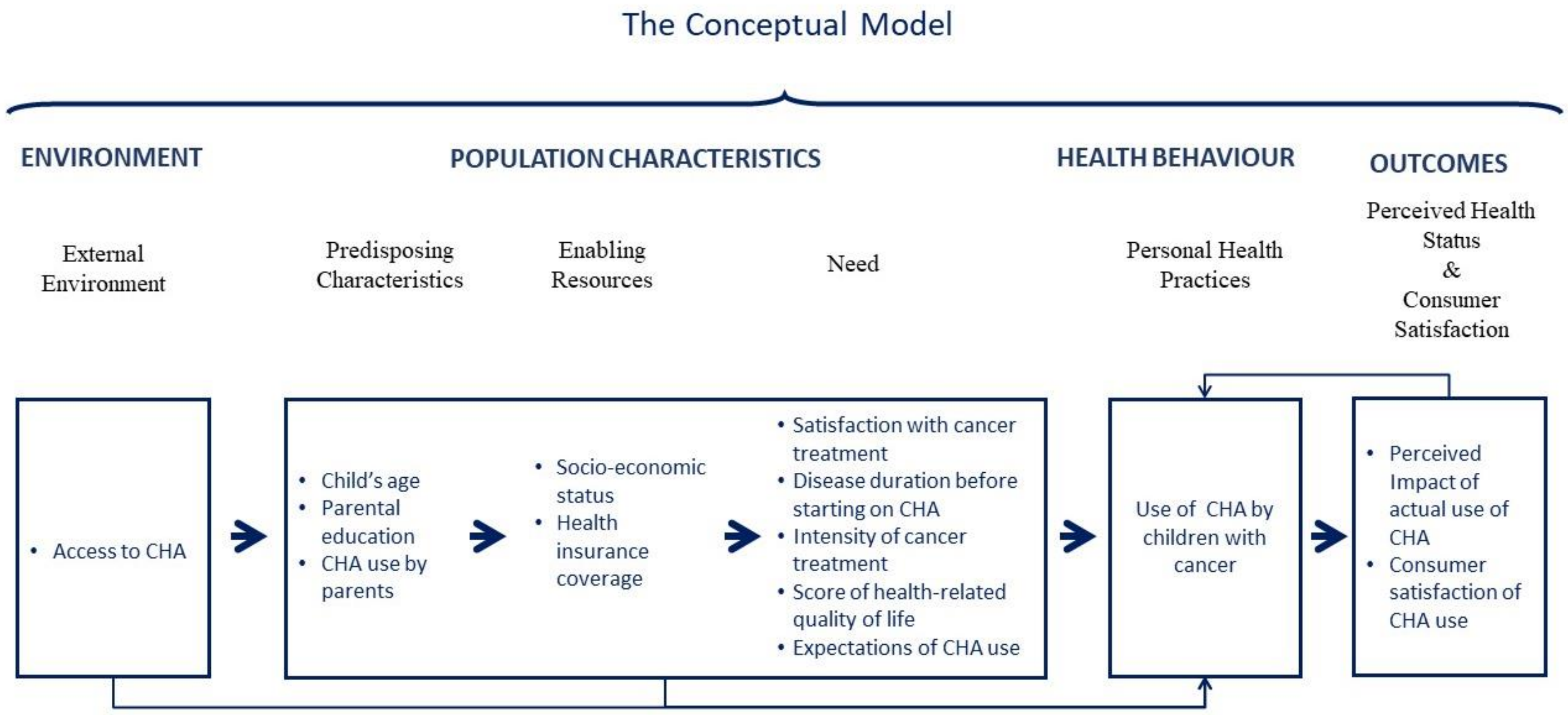

* *Andersen RM. Revisiting the behavioral model and access to medical care: does it matter? Journal of Health and Social Behavior 1995;36(1):1-10.*
