# Supplementary material for: Assessment of complementary health approaches use in pediatric oncology: Modification and preliminary validation of the "Which Health Approaches and Treatments Are You Using?" (WHAT) questionnaires
Source: PLoS One. 2024 Mar 6;19(3):e0294393. doi: 10.1371/journal.pone.0294393 (PMC10917275; doi:10.1371/journal.pone.0294393)
Supplement: S2 Appendix — (PDF) [file pone.0294393.s002.pdf]

The Modified Which Health Approaches and Treatments are you using? (WHAT) - Child Version

This questionnaire asks about the treatments and approaches you may have used for cancer. Some may have been recommended by your health care team, or you may have decided to use them on your own. The goal of this questionnaire is to start a discussion about these treatments with your health care providers.

Section 1: Recommended Treatments by Health Care Team

You may be using treatments that are recommended by your primary health care team for cancer care (for example, nurse, nurse practitioner, doctor, pharmacist, psychologist, physiotherapist, occupational therapist, dietitian) or by your family doctor.

The following list includes types of treatments that could be recommended by your health care team since cancer diagnosis. Please select which treatments were recommended for you?

* select all that apply
* must provide value

- ☐ Chemotherapy by injection (by needle)
- ☐ Chemotherapy by mouth (pills or liquid)
- ☐ Medications by injection (by needle), other than chemotherapy
- ☐ Medications by mouth (pills or liquid), other than chemotherapy
- ☐ Radiotherapy
- ☐ Surgery
- ☐ Bone Marrow Transplant
- ☐ Physiotherapy
- ☐ Occupational Therapy
- ☐ Special Nutrition
- ☐ Psychological Therapy
- ☐ Other

Section 2: Complementary Health Approaches

This questionnaire will ask you questions about other treatments and practices that are known as complementary health approaches.

Here is a list of some examples:

- Nutritional Approaches. Examples include herbs in pills or creams, vitamins, minerals, diets such as gluten- dairy- or glucose-free, special food, juices, or teas
- Cannabis. Examples include dried flower, concentrate, edible oil, smoking/vaping, spray, cannabis in capsules, gummy/candy, or creams.
- Psychological Approaches. Examples include meditation, hypnosis, imagery, prayer, relaxation, mindfulness.
- Physical Approaches. Examples include acupuncture, massage, osteopathy, chiropractic.
- Combinations of Nutritional and Psychological Approaches. Examples include mindful eating.
- Combinations of Physical and Psychological Approaches. Examples include yoga, dance therapy.
- Other Approaches. Examples include homeopathy, aromatherapy, copper bracelets, magnets, reflexology, heating/cooling creams, Chinese Medicine.

Section 2a: Your use of complementary health approaches after cancer diagnosis

Note: All questions on this page ask about your use of Complementary Health Approaches since cancer diagnosis.

1. Have you used complementary health approaches since your cancer diagnosis?

* must provide value

- ☐ Yes
- ☐ No

Why?
* select all that apply
* must provide value

- ☐ Cure cancer
- ☐ Prevent cancer symptoms
- ☐ Treat cancer symptoms
- ☐ Reduce side effects of cancer treatments
- ☐ Feel better
- ☐ It is natural or safe
- ☐ Nothing else worked
- ☐ It was recommended
- ☐ Make my parent(s) or caregiver(s) happy
- ☐ Other reasons

2. Which types of complementary health approaches did you use after your cancer diagnosis?
* select all that apply
* must provide value

- ☐ Nutritional Approaches
- ☐ Cannabis
- ☐ Psychological Approaches
- ☐ Physical Approaches
- ☐ Combinations of Nutritional and Psychological Approaches
- ☐ Combinations of Physical and Psychological Approaches
- ☐ Other Approaches

3. Have you ever talked about complementary health approaches with your health care team?
* must provide value

- ☐ Yes
- ☐ No

4. Who decided that you should use complementary health approaches?
* select all that apply
* must provide value

- ☐ Me
- ☐ My parent(s) or caregiver(s)
- ☐ Member of my health care team
- ☐ Complementary health care provider
- ☐ Someone else
- ☐ Prefer not to answer

5.Have you ever talked with your parent(s) or caregiver(s) about your use of complementary health approaches?
* must provide value

- ☐ Yes
- ☐ No
- ☐ Not sure

6. Have you changed how you follow your cancer treatment because you used complementary health approaches?
* must provide value

- ☐ Yes
- ☐ No
- ☐ Not sure

1. a. Why not?
* select all that apply
* must provide value

- ☐ I believed it would not improve my health
- ☐ I did not have enough information about it
- ☐ The health care team did not recommend it
- ☐ I did not want to use it
- ☐ It costs too much
- ☐ It was difficult to find
- ☐ I was afraid of the side effects or mixing it with my cancer treatments
- ☐ Other reasons

2. Have you ever talked about complementary health approaches with your health care team?
* must provide value

- ☐ Yes
- ☐ No

Move to question #8 (page 4)

Section 2b: Your recent use of Complementary Health Approaches

7. Have you used complementary health approaches in the past four weeks?

* must provide value

- ☐ Yes
- ☐ No

Move to next page

In this section, you will be asked to select each type of complementary health approaches (one by one) that you used during the past four weeks, followed by some questions.

Please select Complementary Health Approach:

* must provide value

- ☐ Nutritional Approaches
- ☐ Cannabis
- ☐ Psychological Approaches
- ☐ Physical Approaches
- ☐ Combinations of Nutritional and Psychological Approaches
- ☐ Combinations of Physical and Psychological Approaches
- ☐ Other Approaches

What were the reasons for using Complementary Health Approach in the past four weeks?

* select all that apply
* must provide value

- ☐ Cure cancer
- ☐ Prevent cancer symptoms
- ☐ Treat cancer symptoms
- ☐ Reduce side effects of cancer treatments
- ☐ Feel better
- ☐ It is natural or safe
- ☐ Nothing else worked
- ☐ It was recommended
- ☐ Make my parent(s) or caregiver(s) happy
- ☐ Other reasons

How helpful was Complementary Health Approach in the past four weeks?

* must provide value

- ☐ Helpful
- ☐ Somewhat helpful
- ☐ Not helpful
- ☐ Not sure

What were the benefits of Complementary Health Approach in the past four weeks?

* select all that apply
* must provide value

- ☐ Feel better
- ☐ Less pain
- ☐ More energy
- ☐ Take fewer prescribed medications
- ☐ Other

What were the risks or disadvantages of Complementary Health Approach in the past four weeks?

* select all that apply
* must provide value

- ☐ Side effects
- ☐ Takes time
- ☐ Takes effort
- ☐ Cost
- ☐ Did not work
- ☐ Other

Have you used more types of complementary health approaches in the past four weeks?

* must provide value

- ☐ Yes
- ☐ No → Move to next page

Please ask for an extra page

Section 2c: About your future use of Complementary Health Approaches

8. Do you plan on using complementary health approaches in the future?

* must provide value

- ☐ Yes
- ☐ No
- ☐ Not sure

Additional Information

Additional information about complementary health approaches that you want your cancer health care providers to know:

____

____

____

____

____

____

____

____
