# Supplementary material for: Assessment of complementary health approaches use in pediatric oncology: Modification and preliminary validation of the "Which Health Approaches and Treatments Are You Using?" (WHAT) questionnaires
Source: PLoS One. 2024 Mar 6;19(3):e0294393. doi: 10.1371/journal.pone.0294393 (PMC10917275; doi:10.1371/journal.pone.0294393)
Supplement: S3 Appendix — (PDF) [file pone.0294393.s003.pdf]

# The Modified Which Health Approaches and Treatments are you using? (WHAT) - Parent Version

This questionnaire asks about the treatments and approaches you may have used for your child's cancer. Some may have been recommended by his/her health care team, or you may have decided to use them for your child on your own. The goal of this questionnaire is to start a discussion about these treatments with your child's health care providers.

## Section 1: Recommended Treatments by Your Child's Health Care Team

Your child may be using treatments that are recommended by his/her primary health care team for cancer care (for example; nurse, nurse practitioner, doctor, pharmacist, psychologist, physiotherapist, occupational therapist, dietitian) or by your child's family doctor.
The following list includes types of treatments that could be recommended by your child's health care team since cancer diagnosis. Please select which treatments were recommended for your child?

* select all that apply
* must provide value

- ☐ Chemotherapy by injection (by needle)
- ☐ Chemotherapy by mouth (pills or liquid)
- ☐ Medications by injection (by needle), other than chemotherapy
- ☐ Medications by mouth (pills or liquid), other than chemotherapy
- ☐ Radiotherapy
- ☐ Surgery
- ☐ Bone Marrow Transplant
- ☐ Physiotherapy
- ☐ Occupational Therapy
- ☐ Special Nutrition
- ☐ Psychological Therapy
- ☐ Other

## Section 2: Complementary Health Approaches

This questionnaire will ask you questions about other treatments and practices that are known as complementary health approaches.

Here is a list of some examples:

- Nutritional Approaches. Examples include herbs in pills or creams, vitamins, minerals, diets such as gluten- dairy- or glucose-free, special food, juices, or teas
- Cannabis. Examples include dried flower, concentrate, edible oil, smoking/vaping, spray, cannabis in capsules, gummy/candy, or creams.
- Psychological Approaches. Examples include meditation, hypnosis, imagery, prayer, relaxation, mindfulness.
- Physical Approaches. Examples include acupuncture, massage, osteopathy, chiropractic.
- Combinations of Nutritional and Psychological Approaches. Examples include mindful eating.
- Combinations of Physical and Psychological Approaches. Examples include yoga, dance therapy
- Other Approaches. Examples include homeopathy, aromatherapy, copper bracelets, magnets, reflexology, heating/cooling creams, Chinese Medicine.

## Section 2a: Your child's use of Complementary Health Approaches since cancer diagnosis

Note: All questions on this page ask about your child's use of Complementary Health Approaches since cancer diagnosis.

**1. Have you used complementary health approaches for your child since he/she was diagnosed with cancer?**

* must provide value

- ☐ Yes → (go to "Why?")
- ☐ No → (go to "1. a. Why not?")

**1. a. Why not?**

* select all that apply
* must provide value

- ☐ I believed it would not improve my child's health
- ☐ I did not have enough information about it
- ☐ The health care team did not recommend it
- ☐ I did not want to use it for my child
- ☐ It costs too much
- ☐ It was difficult to find
- ☐ I was afraid of the side effects or mixing it with my child's cancer treatments
- ☐ Other reasons

**2. Have you ever talked about complementary health approaches with your child's health care team?**

* must provide value

- ☐ Yes
- ☐ No

Move to question #9 (page 4)

**Why?**

* select all that apply
* must provide value

- ☐ Cure cancer
- ☐ Prevent cancer symptoms
- ☐ Treat cancer symptoms
- ☐ Reduce side effects of cancer treatments
- ☐ Feel better
- ☐ It is natural or safe
- ☐ Nothing else worked
- ☐ It was recommended
- ☐ Other reasons

**2. Which types of complementary health approaches did your child use since cancer diagnosis?**

* select all that apply
* must provide value

- ☐ Nutritional Approaches
- ☐ Cannabis
- ☐ Psychological Approaches
- ☐ Physical Approaches
- ☐ Combinations of Nutritional and Psychological Approaches
- ☐ Combinations of Physical and Psychological Approaches
- ☐ Other Approaches

**3. Have you ever talked about complementary health approaches with your child's health care team?**

* must provide value

- ☐ Yes
- ☐ No

**4. Who decided that your child should use complementary health approaches?**

* select all that apply
* must provide value

- ☐ Me or my spouse
- ☐ My child
- ☐ Member of my child's health care team
- ☐ Complementary health care provider
- ☐ Someone else (like a family member)
- ☐ Prefer not to answer

**5. Have you ever talked with your child about his/her use of complementary health approaches?**

* must provide value

- ☐ Yes
- ☐ No
- ☐ Not sure

**6. Have you changed how your child follows his/her cancer treatment because he/she used complementary health approaches?**

* must provide value

- ☐ Yes
- ☐ No
- ☐ Not sure

7. **Have you ever had any difficulty accessing complementary health approaches?**

- ☐ Yes
- ☐ No

## Section 2b: Your recent use of Complementary Health Approaches

### 8. Have you used complementary health approaches for your child in the past four weeks?

* must provide value

- ☐ Yes
- ☐ No

Move to next page

In this section, you will be asked to select each type of complementary health approaches (one by one) that you used for your child during the past four weeks, followed by some questions.

**Please select Complementary Health Approach:**

* must provide value

- ☐ Nutritional Approaches
- ☐ Cannabis
- ☐ Psychological Approaches
- ☐ Physical Approaches
- ☐ Combinations of Nutritional and Psychological Approaches
- ☐ Combinations of Physical and Psychological Approaches
- ☐ Other Approaches

**Did you consult someone to use Complementary Health Approach in the past four weeks?**

* must provide value

- ☐ Yes
- ☐ No

**What were the reasons for using Complementary Health Approach in the past four weeks?**

* select all that apply

* must provide value

- ☐ Cure cancer
- ☐ Prevent cancer symptoms
- ☐ Treat cancer symptoms
- ☐ Reduce side effects of cancer treatments
- ☐ Feel better
- ☐ It is natural or safe
- ☐ Nothing else worked
- ☐ It was recommended
- ☐ Other reasons

**How helpful was Complementary Health Approach in the past four weeks?**

* must provide value

- ☐ Helpful
- ☐ Somewhat helpful
- ☐ Not helpful
- ☐ Not sure

**What were the benefits of Complementary Health Approach in the past four weeks?**

* select all that apply

* must provide value

- ☐ Feel better
- ☐ Less pain
- ☐ More energy
- ☐ Take fewer prescribed medications
- ☐ Other

**What were the risks or disadvantages of Complementary Health Approach in the past four weeks?**

* select all that apply

* must provide value

- ☐ Side effects
- ☐ Takes time
- ☐ Takes effort
- ☐ Cost
- ☐ Did not work
- ☐ Other

**Have you used more types of complementary health approaches in the past four weeks?**

* must provide value

- ☐ Yes
- ☐ No → Move to next page

Please ask for an extra page

## Section 2c: Your child's future use of Complementary Health Approaches

**9. Do you plan on using Complementary Health Approaches for your child in the future?**

* must provide value

- ☐ Yes
- ☐ No
- ☐ Not sure

## Additional Information

**Additional information about your child's complementary health approaches that you want your cancer health care providers to know:**
