# Supplementary material for: Assessment of complementary health approaches use in pediatric oncology: Modification and preliminary validation of the "Which Health Approaches and Treatments Are You Using?" (WHAT) questionnaires
Source: PLoS One. 2024 Mar 6;19(3):e0294393. doi: 10.1371/journal.pone.0294393 (PMC10917275; doi:10.1371/journal.pone.0294393)
Supplement: Attachment — Submitted filename: 1 - Response to Editor.pdf [file pone.0294393.s005.pdf]

Dr. Erik Loeffen
Academic Editor
PLOS ONE

June 12, 2023

Dear Dr. Loeffen,
Thank you for the constructive feedback on our manuscript "*Assessment of complementary health approaches use in pediatric oncology: Modification and preliminary validation of the "Which Health Approaches and Treatments Are You Using?" (WHAT) questionnaires*.

We have carefully reviewed your comments and revised the manuscript accordingly. Please see below for an itemized response to your comments. We hope the paper is now in an acceptable form for publication in *PLOS ONE*. Thank you for the opportunity to revise and resubmit our manuscript.

Sincerely,

Mohammad R. Alqudimat (Corresponding Author)
Ph.D. Candidate - University of Toronto
m.alqudimat@mail.utoronto.ca

Please submit your revised manuscript by Jun 16 2023 11:59PM.

Additional Editor Comments:
First of all I want to congratulate the authors with an interesting an well-conducted study. The authors have incorporated the good suggestions from the reviewers, and as a result of this the paper has improved substantially. Content-wise this paper is ready for acceptance and publication in Plos ONE. Nevertheless before I make a decision to accept I would urge the authors to shorten the manuscript substantially. From what I can see the text body is between 5000 and 6000 words, and although Plos ONE has no strict word limit this feels much too long for a Delphi&interview/validation study. For instance, line 279-300 is essentially "multiple participants did not find the paper format and the use of tables user-friendly, therefore we chose to adjust...". All the results and numbers can either be removed, put into a table or (my preference) put in detail in the supplementary data. I think shortening the manuscript would increase readability in a great manner. (Especially given the nice, large tables there is no need for an abundance of results/numbers in the text.)
For a swift proces, at resubmission I will handle the manuscript personally, sending for peer review would then not be necessary (as we have completed the step already).

Thank you for the comments and suggestions. We have substantially shortened our manuscript by 89 lines (1,339 words)—the revised text body is now 4,491 words. The following is a summary of this revision. All page and line numbers included in our response below refer to the unmarked version of our manuscript without tracked changes (labelled "manuscript"):

1) We have condensed the results section, as suggested, and moved the details to the existing Table 1 and two new Tables 4 and 6.
    a. The details about participants' geographical locations have been removed from the first paragraph of the results (page 11) and moved to Table 1 "Characteristics of participants who participated in the Delphi survey" (page 12).
    b. The findings of the first cycle of the face and content validity study have been condensed, and specific details about comprehensiveness, comprehensibility, and relevance have been moved to a new Table 4 "Findings of the first cycle of the individual interviews regarding the comprehensiveness, comprehensibility, and relevance" (page 18). Only high-level findings have been kept in the text body (page 17).
    c. The section titled "Resulting modified WHAT questionnaires" has been condensed to describe the modified WHAT.
    d. The findings of the second cycle of the face and content validity study have been condensed, and details about comprehensiveness, comprehensibility, and relevance were moved to a new Table 6 "Findings of the second cycle of the individual interviews regarding the comprehensiveness, comprehensibility, and relevance" (page 22). Only high-level findings were kept in the text body (page 21).
2) Minor changes to parts of the design, discussion, limitation, and conclusion have been made. We have condensed or clarified these parts, and no new ideas have been added.
    a. The procedure of Phase 1:
        i. Lines 160-161: The sentence "Children and parents were asked to rate the importance of each domain and item of the child self-report and parent proxy-report versions, respectively" has been changed to "Children were asked to rate the importance of each domain and item of the self-report version, and parents were asked to rate the proxy-report."
    b. The procedure and data analysis of Phase 2:

i. Lines 191-197: Three sentences describing the interview procedure have been reorganized to ease the readability and to reduce the word count. Here is the revised part: "During the interviews, children were asked to read over the child self-report, parents read the parent proxy-report. Children and parents were then asked to describe what they understood about the meaning of the item and whether it was easy to understand (comprehensibility), any essential items were missing (comprehensiveness), and all items and response options were relevant to the questionnaires' purpose. HCPs were asked to review both versions of the WHAT questionnaires and to answer open-ended questions about item relevance and comprehensiveness of the questionnaires."

ii. Lines 217-218: The word "retaining" has been clarified by adding two words, "modifying, removing, or retaining".

c. Discussion:

i. Lines 309-311: The sentence "We introduce the first preliminary face and content validity tested electronic disease-specific CHA questionnaires (i.e., the modified WHAT) that are designed to assess CHA use and initiate clinical dialogue about CHA use in pediatric oncology" has been condensed and simplified to be "We introduce the first electronic disease-specific CHA questionnaires (i.e., the modified WHAT) that have evidence of face and content validity and are designed to assess CHA use and initiate clinical discussions about CHA in pediatric oncology."

ii. Lines 370-372: The sentence has been clarified by adding "Agreement between the self- and proxy-report is not granted unless confirmed by inter-rater reliability testing; for example," to the original sentence "previous research has shown that proxy-report results are not equivalent to the self-report in a study on perceptions of quality of life in children under chiropractic care."

d. Limitations:

i. Lines 392-393: Minor changes to clarify the original two sentences have been made. Here are the edited sentences "In Phase 2, children, parents, and oncology HCPs were recruited to determine the face and content validity of the WHAT. This was because we aimed to test the face and content validity of the WHAT by a sample of the populations who will use the WHAT in clinical settings: the respondents (children and parents), and data users (oncology HCPs who will use the data to initiate clinical discussion)."

ii. Lines 398-401: A few words were replaced to clarify one study limitation. Here is the edited sentence "We were also not able to recruit participants from pediatric oncology newly arrived immigrant populations in Canada; thus, future research should be conducted to ask for their perspectives, and tailor the WHAT content to their CHA use experience."

e. Conclusions:

i. Lines 407-410: Two sentences have been edited to clarify and simplify the points. The edited sentences are shown as "Children, parents, and HCPs agreed on the content of both versions of the WHAT questionnaires after one cycle of Delphi survey. Two iterative cycles of individual interviews and modifications made to the WHAT questionnaires according to suggestions from children, parents and HCPs have helped ensure adequate face and content validity for use in pediatric oncology."

3) No changes to the reference list have been made.
4) The revised version of our manuscript has been formatted using *PLOS ONE's* style requirements.
