# Supplementary material for: Assessment of complementary health approaches use in pediatric oncology: Modification and preliminary validation of the "Which Health Approaches and Treatments Are You Using?" (WHAT) questionnaires
Source: PLoS One. 2024 Mar 6;19(3):e0294393. doi: 10.1371/journal.pone.0294393 (PMC10917275; doi:10.1371/journal.pone.0294393)
Supplement: Attachment — Submitted filename: 1 - Response to Reviewers.docx [file pone.0294393.s004.docx]

Dr. Erik Loeffen

Academic Editor

*PLOS ONE*

February 8, 2023

Dear Dr. Loeffen,

Thank you for facilitating the very constructive peer review of our manuscript "*Assessment of complementary health approaches use in pediatric oncology: Modification and preliminary validation of the "Which Health Approaches and Treatments Are you Using?" (WHAT) questionnaires* ".

We have carefully reviewed the feedback and revised the manuscript accordingly. Please see below for an itemized response to the editor’s and reviewers’ comments. We hope the paper is now in an acceptable form for publication in *PLOS ONE.*

Sincerely,

Mohammad R. Alqudimat (Corresponding Author)

Ph.D. Candidate - University of Toronto

[m.alqudimat@mail.utoronto.ca](mailto:m.alqudimat@mail.utoronto.ca)

*Thank you for submitting your manuscript to PLOS ONE. After careful consideration, we feel that it has merit but does not fully meet PLOS ONE's publication criteria as it currently stands. Therefore, we invite you to submit a revised version of the manuscript that addresses the points raised during the review process.*

*Please submit your revised manuscript by Feb 17 2023 11:59PM. If you will need more time than this to complete your revisions, please reply to this message or contact the journal office at plosone@plos.org. When you're ready to submit your revision, log on to https://www.editorialmanager.com/pone/ and select the 'Submissions Needing Revision' folder to locate your manuscript file.*

*Please include the following items when submitting your revised manuscript:*

- *A rebuttal letter that responds to each point raised by the academic editor and reviewer(s). You should upload this letter as a separate file labeled 'Response to Reviewers'.*
- *A marked-up copy of your manuscript that highlights changes made to the original version. You should upload this as a separate file labeled 'Revised Manuscript with Track Changes'.*
- *An unmarked version of your revised paper without tracked changes. You should upload this as a separate file labeled 'Manuscript'.*
- *If you would like to make changes to your financial disclosure, please include your updated statement in your cover letter. Guidelines for resubmitting your figure files are available below the reviewer comments at the end of this letter.*

*Journal Requirements:*

*When submitting your revision, we need you to address these additional requirements.*

1. *Please ensure that your manuscript meets PLOS ONE's style requirements, including those for file naming. The PLOS ONE style templates can be found at*

*https://journals.plos.org/plosone/s/file?id=wjVg/PLOSOne_formatting_sample_main_body.pdf and*

*https://journals.plos.org/plosone/s/file?id=ba62/PLOSOne_formatting_sample_title_authors_affiliations.pdf.*

2. *Please include your full ethics statement in the 'Methods' section of your manuscript file. In your statement, please include the full name of the IRB or ethics committee who approved or waived your study, as well as whether or not you obtained informed written or verbal consent. If consent was waived for your study, please include this information in your statement as well.*

The revised version of our manuscript has been formatted using *PLOS ONE's* style requirements. We have also added complete REB information, including the full name of the REB, the REB protocol numbers, and more details describing the obtained informed written and implied consent. The details in the manuscript have been alighted to the ethical statement.

**All page and line numbers included in our responses below refer to the unmarked version of our manuscript without tracked changes (i.e., labeled “manuscript”)**

*Reviewers' comments:*

***Reviewer #1:***

*A validated tool for the assessment of complementary health approaches by patients and their family is much needed. The project involved included international providers through the SIOP working group which is commended.*

*Title:*

*The title is too long, suggestion below*

*Assessment of complementary health approaches in pediatric oncology: Modification of the Which Health Approaches and Treatment Are you Using (WHAT) questionnaire*

We appreciate your suggestion, and we considered changing the title accordingly. We condensed the first part of the title using your suggestion. For the second part, we replaced the word "Adaptation" with "Modification", but we kept the phrase "preliminary validation" since this paper is about both modifying and testing the validity of the WHAT questionnaires. The new title is "Assessment of complementary health approaches use in pediatric oncology: Modification and preliminary validation of the "Which Health Approaches and Treatments Are you Using?" (WHAT) questionnaires".

*Keywords: Too many*

We reduced the number of keywords and kept the essential ones (i.e., six keywords).

*Abstract:*

*The abstract should draw in the interest of the reader; this abstract is too wordy.*

*First sentence: Complementary health approaches (CHA) are commonly used by pediatric patients with cancer; however, few health care providers (HCP) inquire regarding the use of CHA for symptom alleviation.*

We followed the *PLOS ONE* submission guidelines and included 300 words in the original abstract. Indeed, we considered your comment and suggestion. We have revised the first sentence according to your suggestion. Also, the other parts of the abstract have been revised and condensed. The revised abstract now includes 289 words (see pages 2 and 3).

*Reframe from using we in the method and results of the abstract and in these sections of the manuscript*

Thank you for the comment. We used the active voice "as much as possible to create direct, clear, and concise sentences" (Section 4.13 of the APA publication manual 7^th^ edition). However, we agree that we overused the active voice in our first submission. Thus, the abstract and the manuscript have been revised in line with your comment.

*The manuscript is too long, needs editing and sentences are run on*

To address this comment, we have substantially shortened our manuscript by 51 lines. This was the net reduction after condensing the method, streamlining the results, and adding further details to the limitation sections (see below for more information). We also improved the presentation of Table 2 (i.e., results of the Delphi survey) by re-grouping the findings. So, the domain ratings have been moved to be presented first, followed by item ratings.

*Design:*

*Suggest you delete: "This article describes"*

*In adapting and validating the WHAT questionnaire, a Delphi survey was conducted. This survey was distributed electronically to pediatric patients with cancer, parents, pediatric oncology HCPs, pediatric complementary care HCPs, and researchers. Phase 1 allowed consensus and adaption of the questionnaire domains and items for use in pediatric oncology. Phase 2 explored the face and content validity of the questionnaire, assessing relevance, comprehensiveness, and comprehension in the context of pediatric oncology.*

As per the reviewer's suggestions, we have revised this paragraph accordingly (see lines 109-114).

*Line 120: states semi-structured interview… using cognitive interviews. This sentence is not clear*

To improve clarity, this sentence has been revised, and the phrase "using cognitive interviews" has been replaced with the phrase "using think-aloud and probing techniques" (lines 117-118) to clarify the two sub-types of cognitive interviewing methods that have been used in Phase 2.

*Line 122: Interviews focused instead of were focused*

As suggested this sentence has been modified accordingly (line 118).

*Line 132 Participants Section*

*A convenience sample*

We have revised this statement (line 131).

*Line 134-5:* *Patient eligibility included children diagnosed with cancer ≥ 3 months, English-speaking, 8-18 years of age, with a history of CHA during cancer therapy.*

These lines have been revised following your suggestion (lines 132-135). We kept one part of the original sentence: "[…] having used at least one type of CHA since cancer diagnosis according to self- or proxy-report." to clarify how we identified the CHA users since this would not be a documented piece of information in the medical record.

*Line 139-143 describing HCP recruitment is not clear and too wordy. It is unclear who comprises "researchers"*

This part includes long names of two groups (i.e., SIOP and PedCAM) that were mentioned for the first time, making it wordy. However, those lines have been revised to enhance clarity (lines 137 to 142). The word "researchers" has been clarified by adding "CHA" to be "CHA researchers" (line 139).

*Paragraph Procedure Line 161. "We" used 5 times in one paragraph and starts 3 of the sentences*

*On the following page, "we" overused and distracting.*

We have modified the writing style for this procedure paragraph and for the following page. The text has been edited according to your comment (see pages 8 and 9), and sentences started with "We" have been minimized significantly throughout the manuscript.

*Line 173: Participants completed a demographic survey and were then asked to suggest modifications in the questionnaire domains and items, as well as recommendations for domains and items that might be added.*

This sentence has been modified according to your suggestion with a slight change to show the correct order of our procedure steps. Here is the edited sentence (lines 167 – 169): "participants were asked to suggest modifications in the domains and items, as well as recommendations for domains and items that might be added and were then asked to complete a demographic survey."

*The data analysis should not include pronouns, just describe how the analysis was conducted*

We revised the data analysis section accordingly. Pronouns have been removed and we shortened this section (see lines 170-172 and 201-219).

*The result section is too long, and the authors have inferred opinions and comments that should not be included in a result section*

*For example, Line 277; These questions would be best for research purposes*

We share your concern that the results section in the first submission was too long. The results section has been shortened significantly.

Like previous studies on instrument validation and following COSMIN guidelines, we used qualitative data as evidence to guide our decisions about keeping, deleting, or modifying the WHAT items. For the Delphi study, quantitative data was described in the results section without including our opinion. For the short answers by participants that included suggestions for adding some domains, we analyzed these short responses and used them as evidence to decide whether to adjust the WHAT content accordingly.

The analysis process for cognitive interviews included transcribing interviews, documenting field notes, summarizing participants' interpretations/understanding of items, identifying face and content validity concerns, and then deciding about each item (see data analysis paragraph on page 10). Also, we aimed to weigh the input from different data sources (i.e., triangulation) and make the decision for each item – whether to retain, omit, or revise. Thus, decision-making about items is considered the final step of our analysis, leading to the final results (i.e., the modified WHAT versions). Therefore, we included how we made the decision to modify the original WHAT in the results section.

*Page 13 the first paragraph is too wordy and hard to follow*

This paragraph has been edited to clarify the numbers and the proportions (see lines 234 – 238). Also, the CHA definition has been revised to improve the reading flow.

*Line 256-258 needs editing for clarification*

*Line 261: The median rating by HCPs was 9 for all domains and approximately (the end of the sentence is not clear) they rated half the items at a 9?*

We appreciate your suggestion. This section has been revised. All findings in the text that overlapped with information found in Table 2 have been removed (see lines 239 to 247). We also reorganized Table 2 to make it easier to follow. First, the ratings of the domains were presented, followed by the ratings of each item.

*Line 270: Only three participants suggested new domains… These suggestions included….*

We appreciate your suggestion. This line has been edited accordingly (lines 239 and 242).

*Line 295: All participants endorsed understanding the content of the question, expect two 10 year old children.*

Thank you for the suggestion. This line has been edited, and extra words have been removed (lines 262-263).

*Line 335: results should not describe that redcap was used for the project*

Agreed, we revised and moved this part about REDCap from the section about the results of the first cycle of Phase 2 to the "Resulting modified WHAT questionnaires" section (page 24). We wanted to highlight that switching from paper-based to electronic versions was done using a research tool (i.e. REDCap). Once the modified WHAT is ready to be used in clinical practice, it will ideally be integrated into an electronic medical record platform to be part of the clinical assessment.

*Line 435 speaks of two children 8 and 9, but previously they were 10. Are these different patients?*

Our response to this question is yes; these are different participants. The two 10 years old children participated in cycle 1 of Phase 2 (see line 262 under cycle 1 findings). In cycle 2, we had a new sample of participants (lines 363-364), of which two children 8 and 9 years old participated (line 377).

*The results in many sections appear repetitive. Last paragraph page 25*

The results sections have been revised and shortened substantially.

***Reviewer #2:***

*Thank you for the opportunity to review this paper. In general, the manuscript is well-written and organized. The topic is important, and the questionnaire is a valuable tool to facilitate discussions among pediatric oncology patients, their parents, and healthcare providers. I have some minor comments.*

*Pg 8. Line 146. The authors state they are targeting a response rate of 17%; however, they don't provide the population size (e.g., the number of members of the organization or children at the Hospital from sick children). What was the denominator? Did they reach their 17% response rate target?*

We appreciate your suggestion. The response rate for each invited group has been added (lines 224-226): "The Delphi survey was completed by 61 participants with response rates per group as 68% (15/22) of children, 59% (13/22) of parents, 24% (10/41) of PedCAM pediatric HCPs and complementary HCPs and researchers, and 30% (23/77) of SIOP oncology HCPs."

*Pg11, line 219. The author states that MA and BW did the content analysis, but BW is not on the list of authors.*

Thank you for the comment. Yes, Brittany Wiles (BW) is not one of the authors. We meant to add the acknowledgement section in the first submission. Thus, the acknowledgment section has been added to the revised version (page 31) and we acknowledged BW efforts in the content analysis.

*Pg 13. Lines 247-252 These percentages add to 99%; who was the other 1%? (The numbers add up, but the percentages do not)*

We agree that this 1% has been missed by rounding up the percentages to zero decimal places. Thus, we re-rounded the numbers to one decimal place. Now, these percentages add to 100% (i.e., 80.3%, 16.4%, 3.3%); see lines 234-238.

*Pg6, line 106 the sentence states: "Both the child- and parent proxy-report versions of the original WHAT questionnaires include four domains: child CAH use." I believe the authors meant to write CHA; please double-check.*

*In table 2, under the communication domain, check spelling; now it reads; "communication about CAH" I think it is CHA.*

Yes, we meant to say "CHA". These typos have been corrected.

*Discussion:*

*The authors interviewed HCP from different regions of the world, but they don't discuss how this tool can be used or the implications it would have in high vs. low-income countries with a difference in survival rates.*

*What about immigrant populations in Canada? Perhaps this is a limitation of the development of the questionnaires. I think this is important to address because, in your study, you argue that it is important to adapt and tailor questionnaires to enrich HCP knowledge about CHA, and you might be missing the input of an often-underserved population that HCP has little knowledge about.*

We agree that testing the WHAT in low-middle-income countries and by a sample of pediatric oncology immigrants in Canada is a limitation of our study and requires future research. This limitation has been added to the limitation section (see lines 488 - 494).

*Also, if HCP from different countries were interviewed, why were only Canadian patients included in the study? Wouldn't cultural differences affect the understanding and interpretations of the questions? This might be something worth addressing in the discussion.*

We only invited international HCPs from the International Society of Pediatric Oncology to participate in a Delphi survey (no interviews) to get diverse perspectives and reach consensus on the domains and items of the WHAT questionnaires that meant to be used in the multi-cultural Canadian pediatric oncology populations. The Delphi survey is not a validity testing study; it is an important step in instrument development/adaptation (i.e., to identify the pool of domains and items that would be tested for validity and reliability). So, the first validity test for the WHAT was done in Phase 2, as we tested the content of the questionnaires in the Canadian context (i.e., no international participants).

The results of the Delphi survey of Canadian children/parents, and Canadian/International HCPs showed that all domains and items of the WHAT reached consensus after one cycle – this might highlight that the original WHAT included the key core set of domains and items that a sample of participants from different cultural backgrounds agreed to include after only one cycle of Delphi survey. Also, this might reflect that we included culturally diverse sample of Canadian children and parents in our study. We added some background details to Tables 1 and 2 to show the ethnic diversity of the children and parents who participated in Phases 1 and 2 (see pages 11-12 and 16-18).

*Why were no complementary HCPs included in phase 2: testing face and content validity of the WHAT questionnaires? Could this be another limitation of the study?*

We agree that including complementary HCPs would enrich our understanding of the important items to include in the WHAT questionnaires. This point has been added to the limitations section (see lines 483-488).
